# SFPQ rescues F508del-CFTR expression and function in cystic fibrosis bronchial epithelial cells

**DOI:** 10.1038/s41598-021-96141-w

**Published:** 2021-08-17

**Authors:** Parameet Kumar, Dharmendra Kumar Soni, Chaitali Sen, Mads B. Larsen, Krystyna Mazan-Mamczarz, Yulan Piao, Supriyo De, Myriam Gorospe, Raymond A. Frizzell, Roopa Biswas

**Affiliations:** 1 Department of Anatomy, Physiology and Genetics, School of Medicine, Uniformed Services University of the Health Sciences, Bethesda, MD 20814 USA; 2 Department of Cell Biology, University of Pittsburgh, School of Medicine, Pittsburgh, PA 1526 USA; 3 Laboratory of Genetics and Genomics, National Institute on Aging, National Institutes of Health, Baltimore, MD 21224-6825 USA; 4 Department of Anatomy, Physiology and Genetics, School of Medicine, Uniformed Services University of the Health Sciences, 4301 Jones Bridge Road, Bethesda, MD 20814 USA

**Keywords:** Cell biology, Molecular biology, Diseases

## Abstract

Cystic fibrosis (CF) occurs as a result of mutations in the cystic fibrosis transmembrane conductance regulator (*CFTR*) gene, which lead to misfolding, trafficking defects, and impaired function of the CFTR protein. Splicing factor proline/glutamine-rich (SFPQ) is a multifunctional nuclear RNA-binding protein (RBP) implicated in the regulation of gene expression pathways and intracellular trafficking. Here, we investigated the role of SFPQ in the regulation of the expression and function of F508del-CFTR in CF lung epithelial cells. We find that the expression of SFPQ is reduced in F508del-CFTR CF epithelial cells compared to WT-CFTR control cells. Interestingly, the overexpression of SFPQ in CF cells increases the expression as well as rescues the function of F508del-CFTR. Further, comprehensive transcriptome analyses indicate that SFPQ plays a key role in activating the mutant F508del-CFTR by modulating several cellular signaling pathways. This is the first report on the role of SFPQ in the regulation of expression and function of F508del-CFTR in CF lung disease. Our findings provide new insights into SFPQ-mediated molecular mechanisms and point to possible novel epigenetic therapeutic targets for CF and related pulmonary diseases.

## Introduction

Cystic fibrosis (CF) is a common life-limiting autosomal recessive genetic disease. This disease occurs as a result of mutations in the cystic fibrosis transmembrane conductance regulator (*CFTR*) gene. There are over 2000 known mutations in CFTR gene, F508del-CFTR being the most prevalent in CF. The *F508del-CFTR* mutation causes the F508del-CFTR protein to misfold, leading to its premature degradation and failure to traffic to the plasma membrane with a consequent loss of a cAMP-activated chloride channel^1^, and activation of massive pro-inflammatory signaling^2–4^. F508del-CFTR functional rescue can be achieved by low temperature^5^ or by small molecules^6,7^, and the drug VX-809^8^. Recently, Trikafta, a triple combination of Elexacaftor-tezacaftor-ivacaftor, has been approved for CF therapy^9^. Despite these advances, there is still a need for developing additional therapeutics for CF.

Coordinated gene regulation and intracellular trafficking are critical mechanisms for transcriptional and post-transcriptional regulation, and localized translation of target messenger RNA (mRNA) in response to genetic, epigenetic, and environmental factors. In addition, RNA-binding proteins (RBPs) have been shown to be key players in controlling gene transcription and post-transcriptional events^10,11^. RBPs also facilitate the correct localization of target mRNAs, sometimes ensuring that mRNAs are transported to specific cytosolic locations for local translation^12–14^. Thus, RBPs offer transcriptional as well as spatiotemporal post-transcriptional regulation including mRNA splicing, polyadenylation, translocation, stability, and translation. Here we have explored the role of splicing factor proline/glutamine-rich (SFPQ), also known as polypyrimidine tract-binding (PTB)-associated splicing factor (PSF), in the regulation of activity of the mutant F508del-CFTR.

SFPQ is an abundant and ubiquitously expressed multifunctional nuclear protein of the Drosophila behavior human splicing (DBHS) protein family^15,16^. It binds to both DNA and RNA and is implicated in the regulation of gene expression pathways, including alternative splicing, transcription, nuclear RNA retention, paraspeckle formation, RNA transport, DNA repair, translation, apoptosis, and response to viral infection^17–23^. The involvement of SFPQ has been demonstrated in several diseases such as neurological^24–26^, immune-metabolic^27^, genetic^28^, and cancer^29,30^. However, whether SFPQ has any role in the regulation of CF lung disease is completely unknown.

Here, we investigated SFPQ expression and subcellular localization in F508del-CFTR CF lung epithelial cell lines (CFBE41o^−^) compared to control cells (16HBE14o^−^). Since SFPQ expression was relatively reduced in CF cells compared to control cells, we examined the impact of SFPQ over-expression in the CFBE41o^−^ CF cells on the expression and function of F508del-CFTR. Furthermore, to explore the molecular mechanism whereby SFPQ restored mutant CFTR expression and function, we performed genome-wide transcriptome profiling (RNA-seq) in CF cells expressing increased levels of SFPQ.

Overall, our data indicate that SFPQ is a central player in regulating CF lung disease. We find that SFPQ is predominantly localized in the nucleus and is downregulated in F508del-CFTR CF lung epithelial cells compared to WT-CFTR control cells. Importantly, restoration of SFPQ expression rescues F508del-CFTR expression and function in CF lung epithelial cells. The cellular transcriptome associated with over-expression of SFPQ suggest that the differentially expressed genes (DEGs) associated with key pathways are involved in sirtuin signaling pathway, remodeling of epithelial adherens junctions, PTEN signaling, mTOR signaling, G1/S checkpoint regulation, PI3K/AKT signaling, ERK/MAPK signaling, G2/M DNA damage checkpoint regulation, Wnt/β-catenin signaling, and NF-κB signaling. These canonical pathways regulated by SFPQ might lead to the identification of novel therapeutic targets for CF.

## Results

### Expression of SFPQ is reduced in F508del-CFTR CF cells compared to control cells

**Figure 1:**
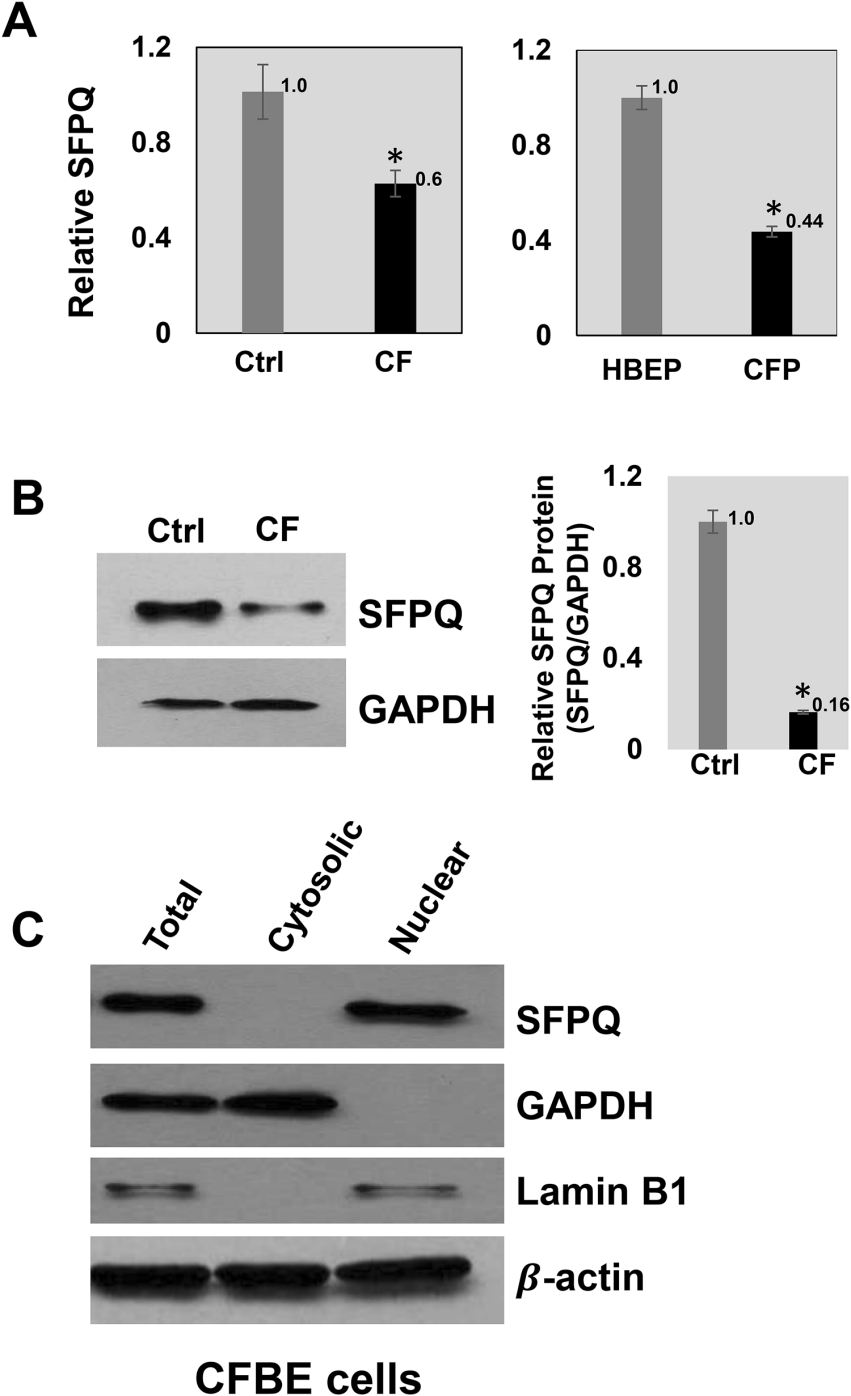
Expression of SFPQ in CF lung cells. (**A**) SFPQ expression was analyzed in RNA isolated from F508del-CFTR CF lung epithelial cell line (CFBE41o^−^) compared to respective control cell line (16HBE14o^−^), and from CF lung parenchyma tissues, and matched control tissues (n = 3, each group). GAPDH was used as an endogenous control. (**B**) SFPQ protein was analyzed by immunoblot in CF and control cells and normalized to GAPDH protein levels. (**C**) Localization of SFPQ protein was analyzed by immunoblot in CFBE cells containing the F508del-CFTR mutation for SFPQ protein localization. The full-length blots are presented in Supplementary materials (Figure S1). The data are representative of two or more independent experiments.

We analyzed the expression of SFPQ in the F508del-CFTR human CF bronchial epithelial cell line (CFBE41o^−^, CF) compared to the respective control cell line (16HBE14o^−^, control) as well as in RNA isolated from lung parenchyma tissues of CF patients undergoing lung transplant (CFP) compared to matched control tissue (HBEP). Interestingly, we find significantly reduced expression levels of *SFPQ* mRNA in CF cell lines (~ 1.6-fold) as well as in CF lung parenchyma tissues (~ 2-fold) compared to respective controls (Figure 1A). Consistently, SFPQ protein was also reduced (~ 5-fold) in CFBE41o^−^ (CFBE) cells compared to 16HBE14o^−^ (HBE) control cells (Figure 1B). This is the first observation for the relative decrease in SFPQ expression in F508del-CFTR CF lung epithelial cells compared to WT-CFTR cells. The reduced expression levels of SFPQ suggests that it may have a role in F508del-CFTR CF lung disease***.***

Subsequently, we analyzed the subcellular localization of SFPQ in CF lung epithelial cell CFBE41o^−^ by immunoblot. We find that SFPQ protein is localized exclusively to the nucleus (Fig. 1C). The full blots are included as supplementary materials (Figure S1).

### SFPQ overexpression increases F508del-CFTR expression in CF cells

**Figure 2:**
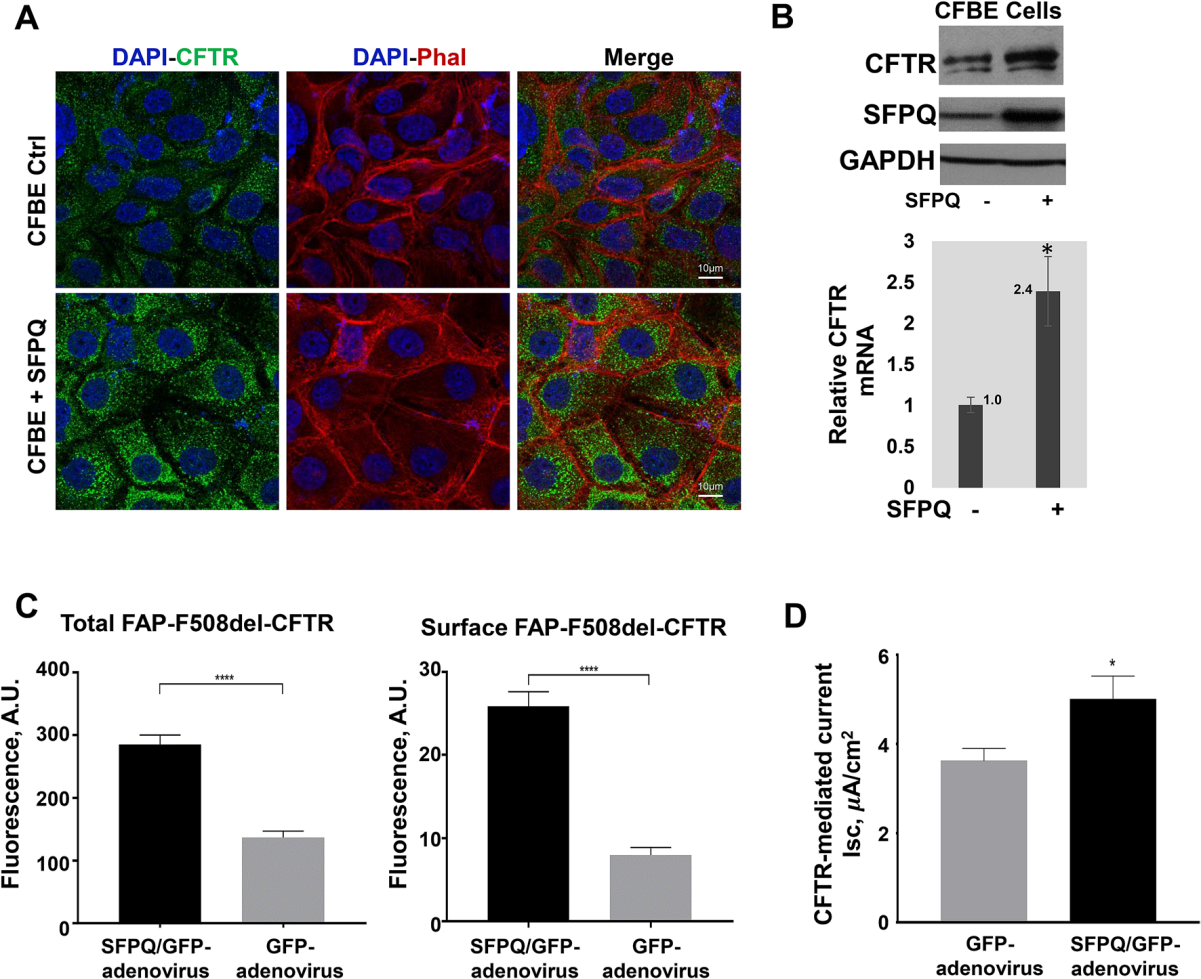
Effect of SFPQ overexpression on CFTR expression. Overexpression of SFPQ in F508del-CFTR CF lung epithelial cell line (CFBE41o^−^) was performed using a vector encoding SFPQ. (**A**) CFBE41o^−^ cells containing the F508del mutation show low levels of CFTR labeling (green) that is faintly perinuclear and punctate. Overexpression of SFPQ in CFBE41o^−^ causes a large increase in the amount of CFTR labeling (green). Green = CFTR, red = phalloidin, blue = DAPI. (**B**) Expression levels of CFTR protein was analyzed by immunoblot and *CFTR* mRNA were measured by RT-qPCR analysis using *GAPDH* mRNA as an endogenous control. (**C**) Total and surface expression of CFTR was analyzed using a FAP assay in F508del-CFTR CFBE410− cells transfected with the adenoviral vector encoding SFPQ with GFP-tag or control vector encoding GFP alone. The graph indicates data normalized from 4 independent experiments. (**D**) F508del-CFTR function was analyzed in CFBE cells by Ussing chamber assays. CFBE cells were cultured at the air–liquid interface (ALI), and after differentiation, they were transfected for 48 h with the adenoviral vector encoding SFPQ with GFP-tag or control vector encoding GFP alone. Forty-eight hours post infection, cells were analyzed for short circuit current. The data are representative of two or more independent experiments.

In order to investigate the role of SFPQ on the expression of F508del-CFTR, we overexpressed SFPQ in CF lung epithelial cells. CFBE41o^−^ cells were transfected with a plasmid vector encoding SFPQ or were mock-transfected. We analyzed the expression of F508del-CFTR in CF lung epithelial cells by immunocytochemistry as well as a fluorogen-based assay. CFBE41o^−^ cells were found to have low levels of F508del-CFTR protein (green) distributed in the cytoplasm and plasma membrane (red) (Figure 2A: top panel). However, when SFPQ was exogenously expressed, a substantial rise in CFTR-specific label (green) was found distributed in a punctate granular pattern in the cytosol, and in regions close to the plasma membrane (red), indicating increased expression and relocation of F508del-CFTR to the apical side of the epithelium. (Figure 2A: lower panel). Yellow fluorescence in the vicinity of the plasma membrane indicates the coincidence of green (CFTR) and red (phalloidin). Consistently, immunoblot analyses of CFTR protein also shows an increased expression induced by over-expression of SFPQ in CFBE cells (Figure 2B: top panel). A corresponding increase in *CFTR* mRNA is also observed (Figure 2B: lower panel). The full blots are included as supplementary materials (Figure S2).

Concurrently, we also performed a fluorogen-based assay to analyze total as well as cell surface expression of fluorogen activated peptide (FAP) tagged F508del-CFTR (FAP-F508del-CFTR). The FAP-F508del-CFBE41o^−^ cells were transduced with adenoviral vector-encoding GFP-tagged SFPQ or control vector encoding GFP alone. Twenty-four hours after transfection, the media was replaced with media containing 5 μM VX-809 to amplify the CFTR surface expression. The cells returned to the incubator for an additional 24 h before commencing the FAP assay. As depicted in Figure 2C, SFPQ induces increased levels of total F508del-CFTR protein with a corresponding increase in surface expression, which further suggested increased expression and relocation of F508del-CFTR to the apical side of the epithelium. Collectively, these data suggest that SFPQ promotes increased expression and relocation of the mutant F508del-CFTR protein to the apical surface of the epithelium in CFBE cells.

To further test for the functional efficacy of SFPQ, CFBE41o^−^ cells were cultured at the air–liquid interface (ALI), and after differentiation, they were transduced for 48 h with the adenoviral vector encoding SFPQ with GFP-tag or control vector encoding GFP alone. Figure 2D shows an Ussing chamber experiment in which SFPQ overexpression induces elevated chloride transport when the cells are exposed to forskolin to elevate cellular cAMP. The increases in current were reduced by addition of the CFTR inhibitor, Inh-172 (Figure 2D). The experiment shown is representative of four independent experiments. These data indicate increased activity of F508del-CFTR CF cells induced by SFPQ, as compared to control cells.

### SFPQ alters transcriptome in CFBE cells

**Figure 3:**
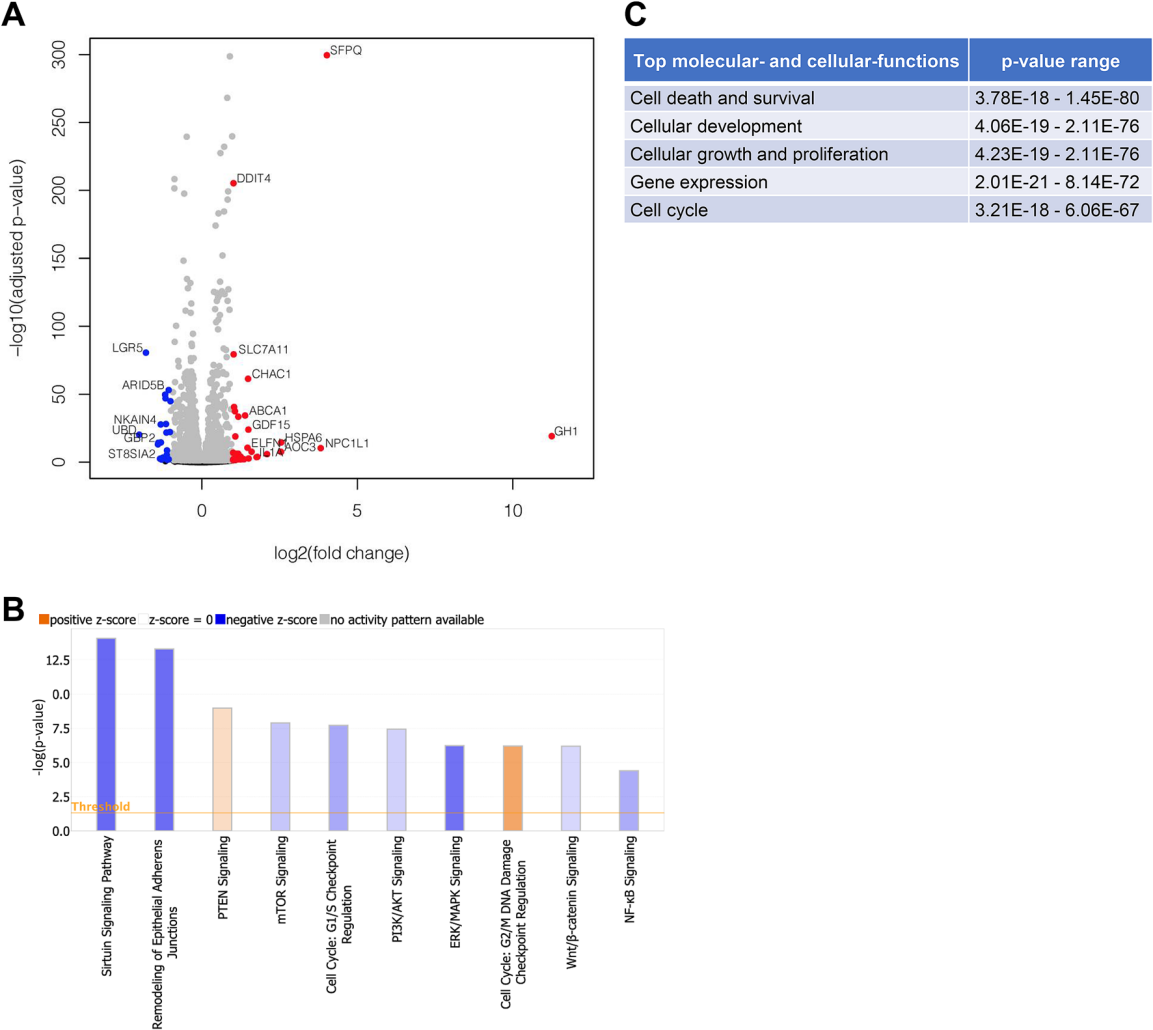
Impact of SFPQ on gene expression profiles and signaling pathways in CFBE cells. (**A**) A volcano plot shows the log2 fold change (x axis) plotted against − log10 (adjusted P value [Padj]) (y axis) of total RNA-sequencing (RNA-seq) data compared between SFPQ overexpressed and control CFBE41o^−^ cells (n = 3 each group). Red circles representing significant upregulated genes, blue circles representing significant downregulated genes and grey color circles representing genes those not meeting the threshold. The statistical criterion for a gene to be considered differentially expressed was an adjusted P value < 0.05, FDR < 0.1, >  ± 2 FC. (**B**) Bar graph shows the differential regulation of canonical signal transduction pathways analyzed by Ingenuity Pathway Analysis (IPA) in the SFPQ overexpressed versus control CFBE41o^−^ cells. The bars indicate the − log (P value) of over-representation, while the orange line indicates the threshold, 2.0. The color of the bars indicates the value of the z-score, as indicated in the legends. (**C**) Top molecular and cellular functions analyzed in the SFPQ overexpressed versus control CFBE41o^−^ cells.

To explore the cellular transcriptome associated with over-expression of SFPQ, we performed genome-wide transcriptome profiling (RNA-seq) analysis in CFBE41o^−^ cells overexpressing SFPQ (n = 3 for each group). The volcano plot shows the log2 (RNA abundance changes in cells overexpressing SFPQ relative to control CFBE41o^−^ cells) and − log10 (adjusted P values) of all RNAs detected by RNA-seq analysis. The statistically differentially expressed genes (DEGs) are depicted in red and blue circles representing significant upregulated and downregulated genes, respectively, and the remaining non-DEGs are plotted as grey circles (Figure 3A). We identified a total of 332 statistically significant DEGs (> 1.5-fold change) in SFPQ over-expressed compared to the control CFBE41o^−^ cells. Among them, 179 (~ 54%) RNAs were upregulated and 153 (~ 46%) RNAs are downregulated in response to SFPQ overexpression. The top differentially expressed RNAs (> 2.5-fold change) in cells overexpressing SFPQ include *GH1*, *NPC1L1*, *HSPA6*, *AOC3*, *CTB-119C2.1*, *BHLHA15*, *IL1A*, *CMYA5*, *BCYRN1*, *GDF15*, *CHAC1*, *ELFN1*, *ABCA*1, *PPP1R32*, and *TNF* mRNA, while those genes that are suppressed by over-expression of SFPQ include *UBD*, *LGR5*, *PIK3IP1*, *GBP2*, *ST8SIA2*, and *NKAIN4* mRNAs*.*

### SFPQ modulates molecular and cellular pathways in CFBE cells

We utilized Ingenuity Pathway Analysis (IPA) software for *in silico* analyses of DEGs in CFBE41o^−^ cells which express increased levels of SFPQ compared to the control CFBE41o^−^ cells. We identified various differentially regulated canonical signaling pathways that are modulated by SFPQ. The top canonical pathways include, sirtuin signaling pathway (p-value = 8.7E−15, z score = − 2.744), remodeling of epithelial adherens junctions (p-value = 5.31E−14, z score= − 2.668), PTEN signaling (p-value = 1.08E−09, z score = 0.7), mTOR signaling (p-value = 1.32E−08, z score = − 1.043), cell cycle: G1/S checkpoint regulation (p-value = 2E−08, z score = − 1.569), PI3K/AKT signaling (p-value = 3.78E−08, z score = − 0.849), ERK/MAPK signaling (p-value = 5.92E−07, z score = − 2.582), cell cycle: G2/M DNA damage checkpoint regulation (p-value = 6.48E−07, z score = 1.789), Wnt/β-catenin signaling (p-value = 6.55E-07, z score = − 0.729), and NF-κB signaling (p-value = 4.83E−05, z score = − 1.511) (Figure 3B).

Consequently, the top ranked molecular and cellular functions significantly affected by SFPQ include cell death and survival (p-value range = 3.78E−18 to 1.45E−80), cellular development (p-value range = 4.06E−19 to 2.11E−76), cellular growth and proliferation (p-value range = 4.23E−19 to 2.11E−76), gene expression (p-value range = 2.01E−21 to 8.14E−72), and cell cycle (p-value range = 3.21E−18 to 6.06E−67) (Figure 3C).

**Figure 4:**
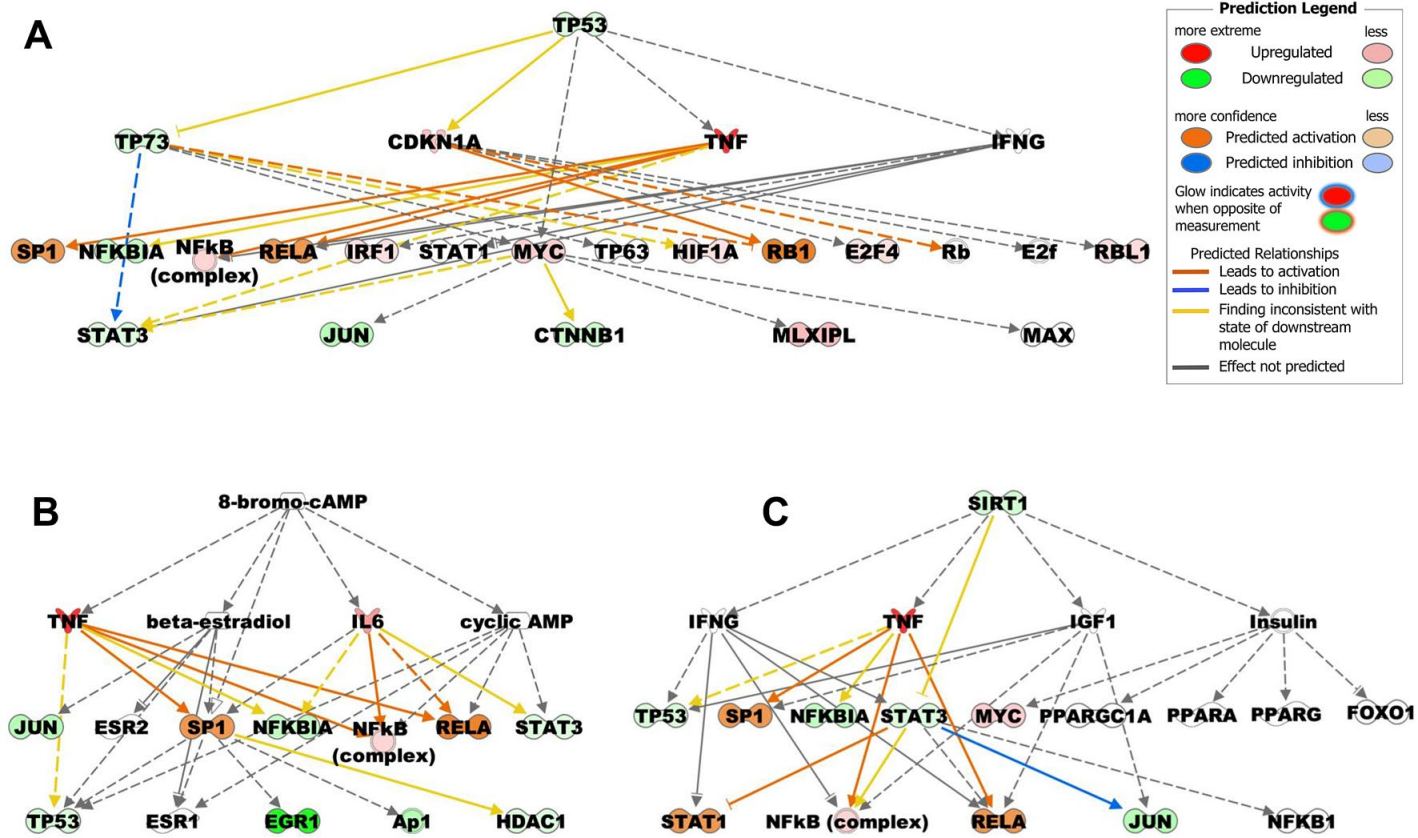
Impact of SFPQ on upstream transcriptional regulators. The charts indicate the most enriched regulatory molecules, (**A**) TP53, (**B**) 8-bromo-cAMP and (**C**) SIRT1, that are predicted to explain the differences in gene expression between SFPQ over-expressed and control CFBE41o^−^ cells. The color of the bars indicates the value of the differential expressions, as indicated in the legends. Solid arrows represent the genes that interact directly, dotted arrows represent indirect interactions between genes.

Further, the upstream regulator analyses suggest the association of several transcriptional regulators with SFPQ. Among those, the topmost regulatory molecules, TP53, 8-bromo-cAMP and SIRT1, that are altered by increased levels of SFPQ in CFBE41o^−^ cells are shown in Figure 4. The corresponding genes are listed in the Supplementary Table 1. These associated molecular and cellular functions likely contributed to increase expression and relocation of F508del-CFTR in CFBE cells after SFPQ overexpression. These results indicate that several molecular and cellular mechanisms and canonical signaling pathways are associated with SFPQ-mediated rescue of F508del-CFTR in CFBE cells.

**Figure 5:**
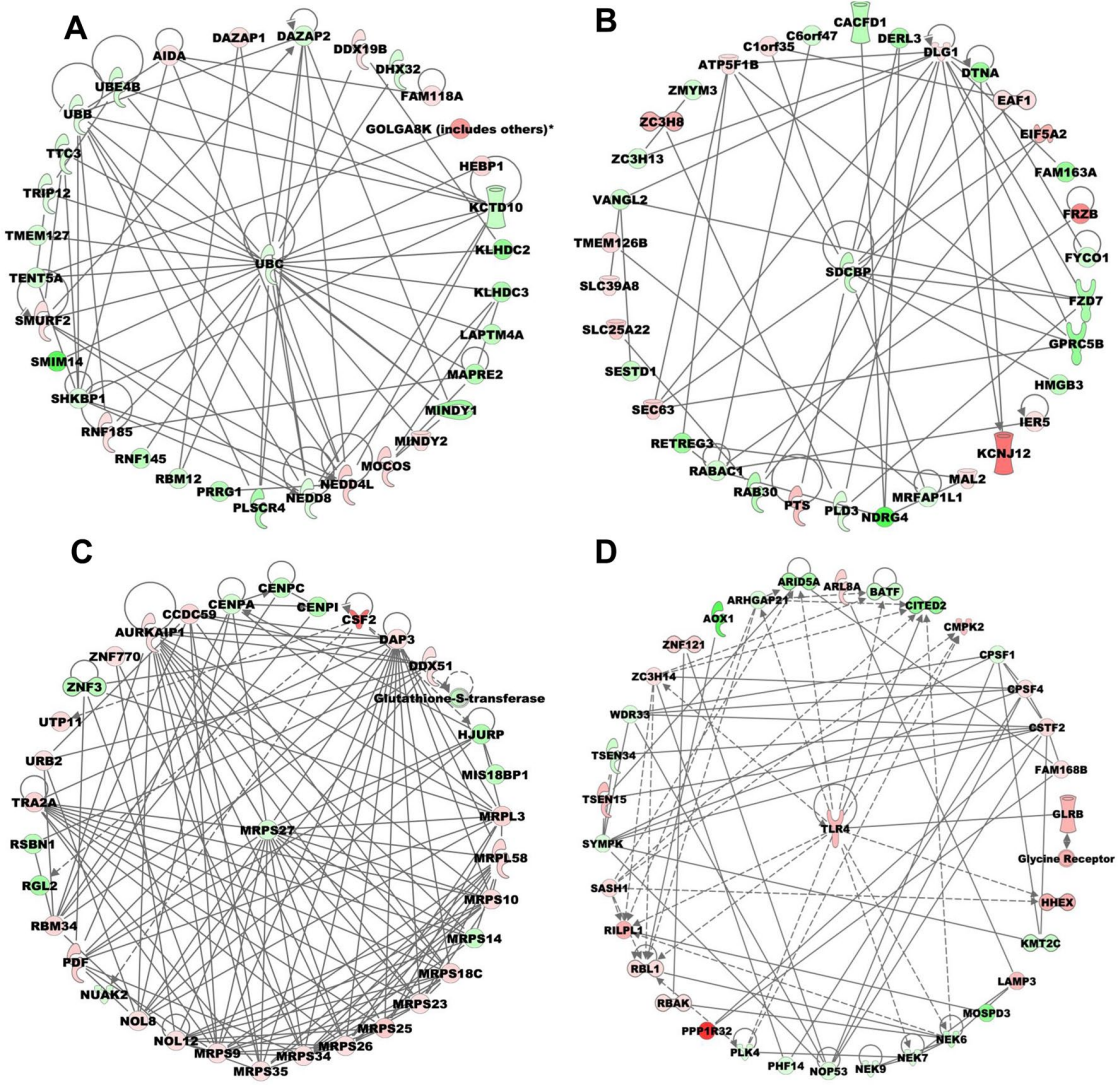
Impact of SFPQ on gene networks. The radial representations indicate the most enriched networks that are predicted to explain the differences in gene expression between SFPQ over-expressed and control CFBE41o^−^ cells. (**A**) Network 1: post-translational modification, protein degradation, protein synthesis; (**B**) Network 2: cell cycle, cell morphology, cellular assembly and organization; (**C**) Network 3: cellular assembly and organization, DNA replication, recombination, and repair, protein synthesis; (**D**) Network 4: cellular function and maintenance, molecular transport, RNA trafficking. Green color denotes low expression or down regulation, red color denotes upregulation whereas grey color denotes those present within our data set but not changed significantly. Solid arrows represent the genes that interact directly, dotted arrows represent indirect interactions between genes.

We further analyzed DEGs to determine regulatory biological relationships mediated by the SFPQ overexpression. The top two major networks with the highest score (31) and focus molecules (35) associated with overexpression of SFPQ are post-translational modification, protein degradation, protein synthesis (Network 1), and cellular assembly and organization, cellular function and maintenance, cellular movement (Network 2) (Figure 5A,B). In Network 1, the genes that are upregulated include *AIDA*, *DAZAP1*, *DDX19B*, *FAM118A*, *GOLGA8K* (includes others), *HEBP1*, *MINDY2*, *MOCOS*, *NEDD4L*, *RNF185*, and *SMURF2*. The genes that are downregulated include *ANXA8*/*ANXA8L1*, *APOBEC3A*, *DAZAP2*, *DHX32*, *KCTD10*, *KLHDC2*, *KLHDC3*, *LAPTM4A*, *MAPRE2*, *MINDY1*, *NEDD8*, *PLSCR4*, *PRRG1*, *RBM12*, *RNF145*, *SHKBP1*, *SMIM14*, *TENT5A*, *TMEM127*, *TRIP12*, *TTC3*, *UBB*, *UBC*, and *UBE4B*. In Network 2, the genes that are upregulated include *ATP5F1B*, *C1orf35*, *DLG1*, *EAF1*, *EIF5A2*, *FRZB*, *IER5*, *KCNJ12*, *MAL2*, *PTS*, *SEC63*, *SLC25A22*, *SLC39A8*, *TMEM126B*, and *ZC3H8*, while genes that are downregulated include *C6orf47*, *CACFD1*, *DERL3*, *DTNA*, *FAM163A*, *FYCO1*, *FZD7*, *GPRC5B*, *HMGB3*, *MRFAP1L1*, *NDRG4*, *PLD3*, *RAB30*, *RABAC1*, *RETREG3*, *SDCBP*, *SESTD1*, *VANGL2*, *ZC3H13*, and *ZMYM3*.

The other networks associated with overexpression of SFPQ with the score (29) and focus molecules (34) are also involved in cellular assembly and organization, DNA replication, recombination, and repair, protein synthesis (Network 3), cellular function and maintenance, molecular transport, RNA trafficking (Network 4), cellular assembly and organization, developmental disorder, DNA replication, recombination, and repair (Network 5), cell signaling, post-translational modification, protein synthesis (Network 6), protein synthesis, RNA damage and repair, RNA post-transcriptional modification (Network 7), cell cycle, organismal injury and abnormalities, RNA post-transcriptional modification (Network 8), drug metabolism, post-translational modification, protein folding (Network 9), and cell cycle, post-translational modification, RNA post-transcriptional modification (Network 10) (Figure 5C,D; Supplementary Figure S3). The corresponding genes are listed in the Supplementary Table 2.

### SFPQ overexpression rescues F508del-CFTR function in CF cells

**Figure 6:**
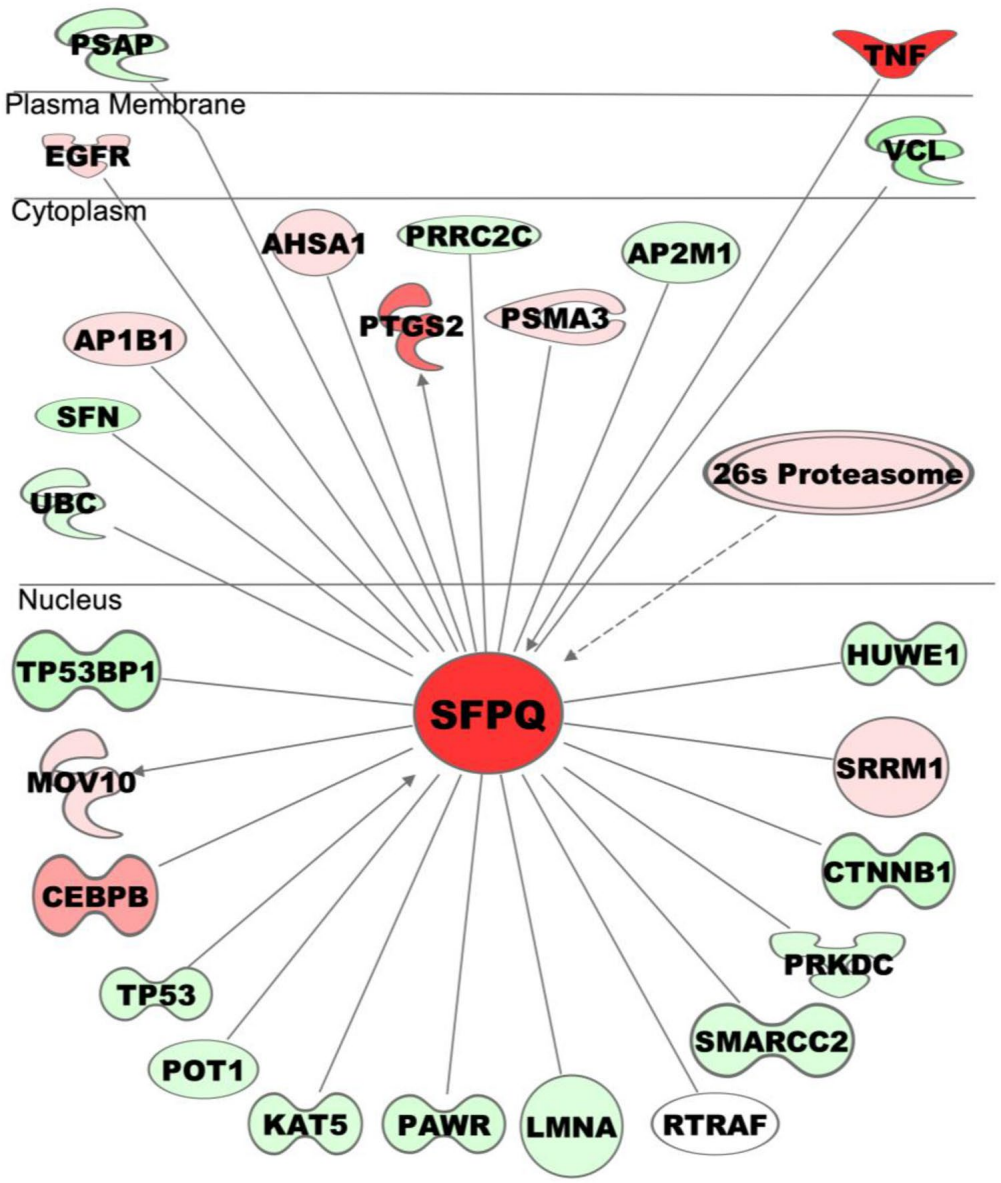
Effect of SFPQ overexpression on CFTR function. The SFPQ focused gene network represents DEGs that are associated with the rescue of CFTR expression, trafficking, maturation and function.

A recent screen of shRNAs on cultured CF epithelial cells have identified multiple genes whose knockdown promoted functional rescue of mutant F508del-CFTR^31^. Our data also identified several of these genes to be differentially expressed in CF cells overexpressing SFPQ. Some of these DEGs have been shown to be associated with SFPQ and linked with CFTR expression, trafficking, maturation and function. As depicted in Figure 6, we found C/EBPß, AHSA1, AP1B1 elevated, and HUWE1 and UBC reduced in SFPQ-overexpressing CF cells. These alterations in gene expression suggest the critical role of SFPQ in various cellular and metabolic pathways that are associated with CFTR processing and trafficking.

## Discussion

CF lung disease is characterized by abnormal chloride transportation and profound inflammatory phenotype, due to mutations in the CFTR gene, the most frequent being deletion of F508. The resulting mutant CFTR protein fails to traffic to the plasma membrane. This study demonstrates that SFPQ is a central player in regulating mutant F508del-CFTR in CF lung disease. Our data indicate that nuclear-localized SFPQ is downregulated in F508del-CFTR CF lung epithelial cells compared to WT-CFTR control cells. This is the first report of an aberrant reduction in SFPQ protein levels in CF lung epithelial cells.

SFPQ belongs to the DBHS family of proteins that include non-POU domain-containing octamer-binding protein (NONO/p54nrb) and paraspeckle protein component 1 (PSPC1) and known to be localized in the nucleus^15,16^. It is a multifunctional RBP and regulates a wide range of cellular processes, including pre-mRNA splicing, transcription, nuclear RNA retention, paraspeckle formation, RNA transport, DNA repair, translation, apoptosis, and response to viral infection^17–23^. Though the functional impact of SFPQ has been shown in various diseases such as neurological^24–26^, immune-metabolic^27^, genetic^28^, and cancer^29,30^, there is no prior report of changes in the SFPQ expression level in CF diseased state. Here, we find reduced levels of nuclear-localized SFPQ protein in F508del-CFTR CF lung epithelial cells. Previously, the downregulation of SFPQ is shown in Alzheimer’s disease (AD) patient’s brains, which contributes to the rapid progression of AD^26^. Our finding that in F508del-CFTR CF lung epithelial cells the level of SFPQ is aberrantly reduced suggests that it may play a role in CF lung disease.

Importantly, our data indicate that exogenous expression of SFPQ in CF lung disease can rescue mutant CFTR expression and function. Not only does increased levels of SFPQ promote increased expression of mutant CFTR, both at mRNA and protein levels, but it also drives more of the mutant CFTR protein to the plasma membrane and rescues the function of F508del-CFTR in CF lung epithelial cells. Previously, it has been shown that SFPQ is a prerequisite for colocalization and coordinated axonal trafficking of transcripts (e.g., *Lmnb2* and *Bcl2l2* mRNAs) to promote axon survival^23^. The depletion of SFPQ in the mouse brain resulted in aberrant processing of neuronal genes characterized by long introns^32^. Further, in another study, depletion of SFPQ decreased proliferation and induced S-phase arrest and apoptosis in BRAF^V600^^E^-driven colorectal and melanoma cells^29^. Our findings indicate the role of SFPQ in regulating transcriptional and post-transcriptional mechanisms as well as intracellular trafficking for F508del-CFTR in CF lung epithelial cells.

Chloride transport into the lumen of the airways is mainly performed by cAMP-activated CFTR and calcium ion-activated chloride channels^33^. However, in CF disease mutations in CFTR, results in the concomitant loss in the function of a cAMP-activated chloride channel. Notably, we find that the impact of SFPQ in CF lung disease is further extended to the rescue of cAMP-activated chloride conductance. Earlier, the role of SFPQ has been shown as a transcriptional activator of phosphodiesterase 3A (PDE3A), a member of the cGMP-inhibited cyclic nucleotide phosphodiesterase (PDE) family^34^. Our results suggest a role of SFPQ in the rescue of CFTR function.

Further, we analyzed SFPQ-mediated genome-wide transcriptome changes in CF lung epithelial cells and mainly focused on those genes that might have a role in CFTR processing and trafficking. Notably, transcriptome profiling of SFPQ overexpressed CFBE41o^−^ cells compared to control CFBE41o^−^ cells reveal several DEGs; the top (> 2.5-fold) changes by SFPQ overexpression include *GH1*, *NPC1L1*, *HSPA6*, *AOC3*, *CTB-119C2.1*, *BHLHA15*, *IL1A*, *CMYA5*, *BCYRN1*, *GDF15*, *CHAC1*, *ELFN1*, *ABCA*1, *PPP1R32*, and *TNF* mRNAs. While those genes that are suppressed by overexpression of SFPQ include *UBD*, *LGR5*, *PIK3IP1*, *GBP2*, *ST8SIA2*, and *NKAIN4*. Adjunctive GH1 therapy as nutritional augmentation in children with cystic fibrosis is in phase 3 clinical trial as it significantly improves height, weight, bone mineral content, lean tissue mass, and decreased number of hospitalizations^35–39^. The role of NPC1L1, Niemann-Pick Type C1-Like 1 transporter, has been shown important for the maintenance of cholesterol homeostasis^40^. Among various heat-shock protein, HSPA6 interacts with misfolded protein and facilitate correct folding and prevent aggregation^41^. The importance of *BCYRN1*, a long non-coding RNA, is determined in asthma, where the increased expression of *BCYRN1* is associated with elevated expression of transient receptor potential channel 1 (TRPC1) that lead to improved transmembrane transport of Ca^2+^ and proliferation of rat airway smooth muscle cell^42^. *CHAC1*, ChaC glutathione-specific γ-glutamylcyclotransferase 1 is a part of the unfolded protein response (UPR) pathway and capable of hydrolyzing reduced form of glutathione (GSH), in CF homeostasis of GSH is faulty^43,44^. Further, it has been found that upregulation of CHAC1 in CF cells prevents the production of IL-8 and CXCL1^45^. A role of ABCA1 has been proposed as a regulator protein of substrate and ion transport, an analogy to CFTR^46^. Further, its crucial role is shown in providing cellular homeostasis of cholesterol and phospholipid^47^. The role of overexpressed TNFα has been shown in enhanced CFTR expression and maturation of F508del-CFTR as well as induction of CFTR chloride currents^48^. UBD is an inflammation maker, whose upregulation promotes inflammation in CF^49^. The elevated expression of LGR5 and its associated Wnt/β-catenin signaling has been demonstrated in CF intestinal crypts^50^.

Beside these considerations, transcriptome profiling of CF cells with increased expression of SFPQ compared to control cells also identified several DEGs that have been previously shown to associate with SFPQ and in separate studies, these genes are linked with CFTR expression, trafficking, maturation and function. For example, we find upregulation of *C/EBPß*, *AHSA1*, *AP1B1*, and downregulation of *HUWE1*, *UBC* in SFPQ overexpressed CF cells. In agreement with our study, it has been demonstrated that C/EBPß, a member of the CCAAT family, binds to the CFTR promoter and positively regulates its transcriptional activity^51–54^. AHSA1 (activator of 90 kDa heat shock protein ATPase 1 or Aha1) is involved in CFTR interactome and is a co-chaperone of HSP90 and regulates its ATPase activity^55,56^. HSP90 facilitates complete folding of wild type(WT)-CFTR, while in the case of F508del-CFTR, HSP90 along with Hsp40/Hsp70 promote proteasome degradation of unstable and misfolded CFTR^55–58^. HUWE1 (E3 ubiquitin protein ligase) is found to regulate ABCG1 and ABCG4 at the post-translational level and participate in cellular cholesterol homeostasis^59^. UBC functions in the proteasome-mediated degradation of unstable and misfolded CFTR, maturation of CFTR and intracellular trafficking of proteins^60,61^.

Further analyses demonstrate that several molecular and cellular functions are differentially regulated, where the topmost affected functions include cell death and survival, cellular development, cellular growth and proliferation, gene expression and cell cycle. Interestingly, in network analyses, we find top networks with the highest score and focus molecules mainly consist of genes required for cellular function and maintenance pathways such as DNA replication, recombination, and repair, RNA damage, repair, post-transcriptional modification, and trafficking, protein degradation, synthesis and folding, and molecular transport. Moreover, in upstream regulator analyses, the gene expression dataset with the increased levels of SFPQ in CFBE41o^−^ cells also revealed the significant association of several transcriptional regulators, wherein the topmost regulatory molecules are TP53, 8-bromo-cAMP and SIRT1. TP53 participates in the cellular stress response, cell division, and DNA repair. 8-bromo-cAMP is contributing to cell proliferation, apoptosis, differentiation and growth. SIRT1 is well-documented in the regulation of pulmonary fibrosis^62,63^. These SFPQ-mediated modulations in gene expression to regulate molecular and cellular-functions likely contributed to increasing expression, relocation and restore the function of F508del-CFTR in CFBE cells.

Subsequently, canonical pathway analyses also identified various differentially regulated signaling pathways such as sirtuin signaling pathway, remodeling of epithelial adherens junctions, PTEN signaling, mTOR signaling, cell cycle: G1/S checkpoint regulation, PI3K/AKT signaling, ERK/MAPK signaling, cell cycle: G2/M DNA damage checkpoint regulation, Wnt/β-catenin signaling and NF-κB signaling. These results, activation or inhibition of canonical signaling pathways in SFPQ overexpressed CFBE cells, are in agreement with earlier studies. Likewise, autophagy plays a critical role in the removal of misfolded or aggregated proteins and PI3K/Akt/mTOR signaling pathway has been shown to have a vital role in balancing cellular proteostasis and autophagy^64^. In CF, defective autophagy is one of the causes for the formation of F508del-CFTR aggresome^65^. In agreement with our results that PI3K/AKT and mTOR signaling are reduced, suppression of the PI3K/AKT/mTOR signaling pathway enhances CFTR stability and expression via reinstating autophagy in F508del-CFTR CFBE41o^−^ cells^66^. Further, our results show that ERK/MAPK signaling is inhibited after SFPQ expression, which is in line with the previous report that identified increased expression of ERK/MAPK in F508del-CFTR cells^67^. In CF, the consequence of F508del-CFTR mutation in lung epithelial cells is well documented in enhanced activation of NF-κB signaling, which is associated with the CFTR dysfunction i.e., incompetence of F508del-CFTR to reach cell surface^68–70^. Further, in accordance with our study, NF-κB signaling is inhibited by SFPQ overexpression, the downregulation of NF-κB has been shown through the expression of functional CFTR on the cell surface in CFBE4lo^−^ cells^70,71^. The graphical summary of IPA-based analyses also predicted that increased expression of SFPQ in CFBE41o^−^ cells lead to activation of several key functions such as binding of DNA, synthesis of lipid, stimulation of cells, proliferation of epithelial cells, growth of connective tissue and adhesion of myeloid cells (Supplementary Figure S4). Collectively, our results suggest that the SFPQ-mediated F508del-CFTR rescue mechanism is wide and multifaceted, and is possibly involving several molecular and cellular mechanisms and canonical signaling pathways.

In closing, we report for the first time an aberrant reduction of SFPQ in a CF diseased state and participation of SFPQ in the rescue of expression and function of F508del-CFTR in CF lung disease. Our data is uncovering a potential mechanism of suppressed chloride conductance in CF cells. These data provide new insights into epigenetic mechanisms mediated by SFPQ function in CF lung disease. This study may also serve as a paradigm for similar complex regulatory mechanisms in other pulmonary disorders. Further exploration of the SFPQ-regulated mechanisms by which SFPQ regulates cAMP-activated chloride conductance, and what causes suppression of SFPQ in CF are important aspects for future studies. SFPQ is known to recruit epigenetic silencers such as Sin3A and HDAC to the promoter of target genes^16^. These factors have also been shown to be important players in the rescue of CFTR function^72,73^. Moreover, SFPQ is part of the CFTR-interactome, acting in concert with poly-pyrimidine tract-binding protein (PTBP1)^74^. Thus, multiple processes, in which SFPQ plays a central role, are operating in concert to regulate CF lung diseases, characterized by inflammation, fibrotic factor as well as lack of functional CFTR. Understanding these mechanisms will lead to novel epigenetic therapeutic targets for CF and related pulmonary diseases.

## Material and methods

### Reagents and antibodies

The following reagents were used: MEMα (Gibco, 12571-048), 0.05% Trypsin–EDTA (Gibco, 25300-054), 0.25% Trypsin–EDTA (Gibco, 25200114), Puromycin (Gibco, A1113803), Fetal Bovine Serum (Gibco, 10437-010), Opti-MEM (Gibco, 31985-062), Lipofectamine 2000 transfection reagent (Invitrogen, 11668019), Pen Strep (Gibco, 15140-122) miRVana kit (Ambion, AM1560). Antibodies against the following proteins were used as recommended by the manufacturers: GAPDH (Millipore, MAB374), C-terminal CFTR antibody, clone 24-1 (R&D systems, MAB25031), for surface expression of CFTR (abcam, ab2784), SFPQ (abcam, ab177149 and Bethyl Laboratories A301-321A), Lamin B1 and β-actin (Cell Signaling, 13435 & 8H10D10 respectively). All primary antibodies were used at 1:1000 dilution. SFPQ (untagged)-Human splicing factor proline/glutamine-rich (SFPQ) cDNA was purchased from Origene (SC127926). Taqman assay for candidate genes SFPQ (ID: Hs00192574_m1) and CFTR (ID: Hs00357011_m1) was used to analyze mRNA expression and was obtained from Life technologies.

### Cell culture

The CFBE cells (CFBE41o−) which stably expressed F508del-CFTR (CFBE-F508del) and WT-CFTR 16HBE14o− (HBE) cells were purchased from Millipore Sigma and cultured following the manufacturer’s protocol. All cells were maintained in a humidified 5% CO_2_, 95% air incubator at 37 °C. For SFPQ overexpression 2.5 × 10^5^ cells were plated in a 6-well plate. CF cells were transfected with a vector encoding SFPQ (500 ng) using lipofectamine 2000.

### Human specimen

Lung parenchyma tissues from six subjects undergoing lung transplant were obtained for this study by the University of Pittsburgh Cystic Fibrosis Research Center: 3 CF and 3 matched non-CFs. The tissues were collected from each of these individuals under an IRB protocol approved by the University of Pittsburgh Institutional Review Board. All methods were carried out in accordance with relevant guidelines and regulations and informed consent to participate were obtained. No organs or tissues were procured from prisoners.

### RT-PCR

Total RNA extraction, cDNA synthesis, and gene expression were performed using mirVana miRNA Isolation Kit, High Capacity cDNA Reverse Transcription Kit (Applied Biosystems, 4368813), and TaqMan gene expression assay, respectively, accordingly the manufacturer's instructions. The 2^*−ΔΔCT*^ method was applied for the calculation of relative fold changes, where normalization of real-time PCR data was attained against β-actin (an endogenous control)^75^.

### Western Blotting

Standard methods were used for protein extraction and immunoblot analysis. Briefly, RIPA buffer plus protease/phosphatase inhibitors (Sigma P5726 and S8820, respectively) and Tris–Glycine gel (4–12%) were used for the lysis of cells and electrophoresis of samples (50 μg protein), respectively, as described previously^77^, with some modifications. Dilution of 1:1000 was applied for all primary antibodies.

### Immunocytochemistry

CFBE cells were fixed and stained as previously described in Kumar et al.^76^, with minor modification. CFBE cells were seeded onto a coverglass, precoated with poly-d-lysine hydrobromide (50 μg/ml) (Sigma, P7886). The cells were washed twice with phosphate-buffered saline (PBS) and then fixed for 15 min with 4% paraformaldehyde at room temperature. Fixative solution was aspirated and cells were washed three time with 1 × PBS for 5 min. Cells were then permeabilized with 0.2% triton X-100 for 10 min at room temp. Permeabilized cells were again washed with sterile 1× PBS, for 5 min and then blocked with blocking buffer (1% BSA + 0.3% triton X-100 in 1× PBS). Primary antibodies (mouse anti-CFTR, 1:500, R&D Systems and Rabbit anti- SFPQ, 1:750, Bethyl Laboratories), diluted in blocking buffer, were added to the cells and incubated overnight at 4 °C. Next day cells were washed and incubated for 2 h at room temperature in appropriate secondary antibodies. After washing, the cells were then incubated in DAPI for 15 min and the cover glass was mounted with Fluoromount-G (Invitrogen, 00-4958-02). Samples were imaged using a Zeiss 700 confocal microscope and an average of 300 cells in 10 different fields was analyzed.

### Expression of functional cell surface protein

CFBE cells stably expressing a FAP-F508del CFTR construct were generated as described for a 293A stable cell line^77^. The FAP-F508del CFBE cells were plated in a 96-well plate in complete DMEM containing 8 µg/ml polybrene with or without addition of recombinant adenovirus encoding SFPQ/GFP or GFP alone. The day after plating, the growth media was replaced by complete DMEM containing 5 µM VX-809. VX809 was used to amplify the CFTR surface expression for more reliable detection. The following day, FAP-F08del CFTR surface expression was detected quantitatively by automated microscopy analysis as described previously^77^.

### Ussing chamber assay

Costar Transwell filters (6.5 mm) were used for culturing of F508del-CFTR CF cells and transduction with SFPQ/GFP adenoviral vector or control GFP vectors was performed on the basolateral side of the cell monolayer at 37 °C for 48 h. These filters were then mounted and equilibrated in Physiologic Instruments Ussing chambers as previously described^78^, with some modifications. In the subsequent step, 10 µM amiloride was added to the apical chamber to inhibit epithelial sodium channel (ENaC)-mediated sodium absorption. After 2 min, 10 µM forskolin was added to both the basolateral and apical chambers to activate CFTR-mediated anion excretion. Following another 2 min, CFTR-mediated anion excretion was inhibited using a CFTR Inhibitor-172 (Sigma-Aldrich) to the apical chamber. During this time, currents had achieved steady-state. Short-circuit current (Isc) and transepithelial resistance (TER) were continuously measured using a Physiologic Instruments VCC-MC8 and Physiologic Instruments Acquire and Analyze 2.3 data acquisition hardware and software. Agonist- or inhibitor-induced changes in short-circuit current (ΔIsc) were calculated from differences in the mean Isc over the 10 s period preceding reagent additions.

### Sequencing

Total RNA was extracted miRVana RNA extraction kit (Life Technologies, Thermo Fisher). The quality and quantity of RNA was checked using Agilent RNA 6000 nano kit on the Agilent Bioanalyzer. 400 ng of high-quality RNA was used for sequencing library prep using Illumina TruSeq Stranded mRNA Library prep kit according to manufacturer’s protocol (Illumina, San Diego, CA). The quality and quantity of sequencing libraries were checked using Agilent DNA 1000 kit on the Agilent Bioanalyzer. Single-read sequencing was performed for 141 cycles in Illumina HiSeq 2500 sequencer and Real-Time Analysis (RTA) generated the base-call files (BCL files).

BCL files were de-multiplexed and converted to standard FASTQ files using bcl2fastq program (v2.20.0.422). FASTQ files were trimmed for adapter sequences using Cutadapt version v1.18 and aligned to human genome hg19 Ensembl v82 using STAR software v2.4.0j. featureCounts (v1.6.4) were used to create gene counts from 12 samples. Differential expression analysis of the gene counts was carried out with Bioconductor package DESeq2 version 1.26.0^79^ in R (version 3.6.3). Transcripts were accepted as differentially expressed if they met statistical significance Benjamini–Hochberg adjusted p-value < 0.05 and absolute log2 fold change > 1.

Ingenuity Pathways Analysis (IPA, QIAGEN Inc.) was used to perform functional analysis of the differentially expressed genes.

### Statistical data analyses

Statistical analysis was performed using Excel. Significance values (p ≤ 0.05) were determined by student’s t-test. Error bars on graphs represent SEM.

### Disclaimer

The views expressed are those of the authors and do not reflect the official policy or position of the Uniformed Services University of the Health Sciences, the Department of the Defense, or the United States government.

## Supplementary Information

Supplementary Information.

## Data Availability

The datasets generated during and analyzed during the current study are available from the corresponding author on reasonable request.
