# Supplementary material for: SFPQ rescues F508del-CFTR expression and function in cystic fibrosis bronchial epithelial cells
Source: Sci Rep. 2021 Aug 17;11:16645. doi: 10.1038/s41598-021-96141-w (PMC8371023; doi:10.1038/s41598-021-96141-w)
Supplement: Supplementary file 1 — Supplementary Information. [file 41598_2021_96141_MOESM1_ESM.pdf]

Supplementary material for the manuscript:

SFPQ rescues F508del-CFTR expression and function in cystic fibrosis bronchial epithelial cells

Parameet Kumar, Dharmendra Kumar Soni, Chaitali Sen, Mads B Larsen, Krystyna Mazan-Mamczarz, Yulan Piao, Supriyo De, Myriam Gorospe, Raymond A. Frizzell, and Roopa Biswas

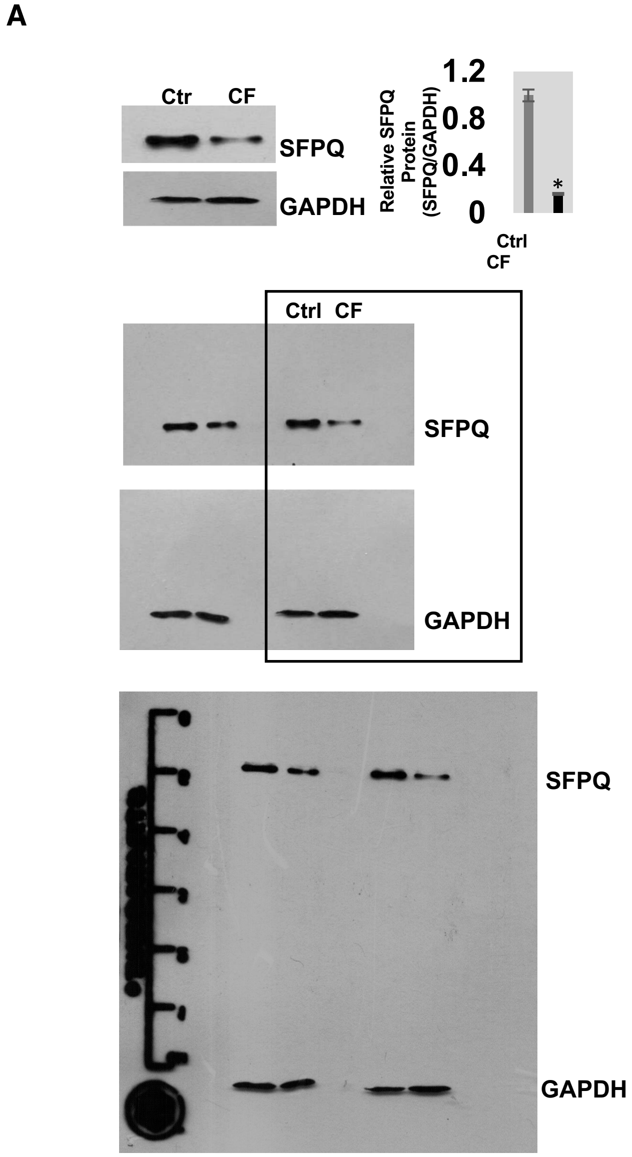

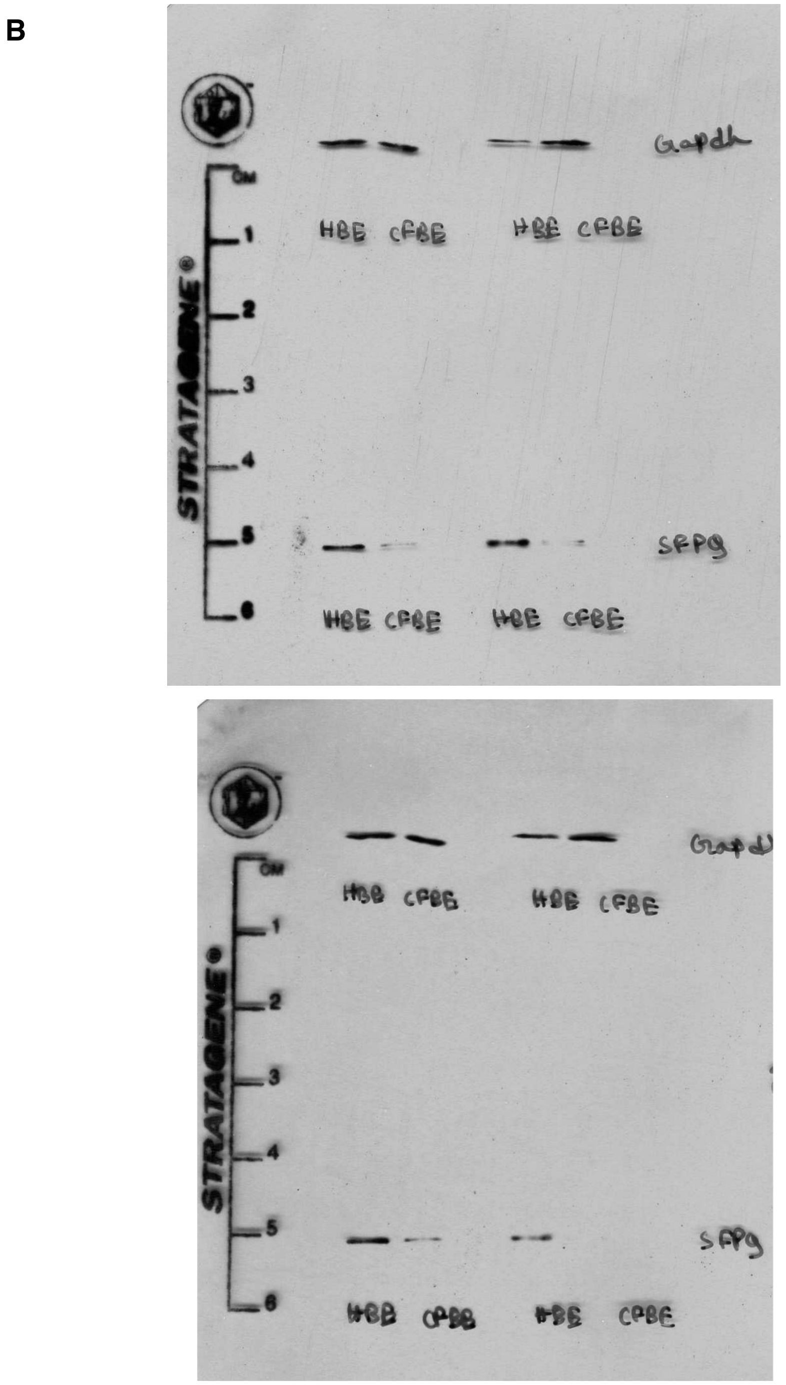

Figure S1: Expression of SFPQ protein in CF lung cells. (A) SFPQ protein was analyzed by immunoblot in CF and control cells and normalized to GAPDH protein levels. **(B)** Multiple exposure s and duplicates are indicated.

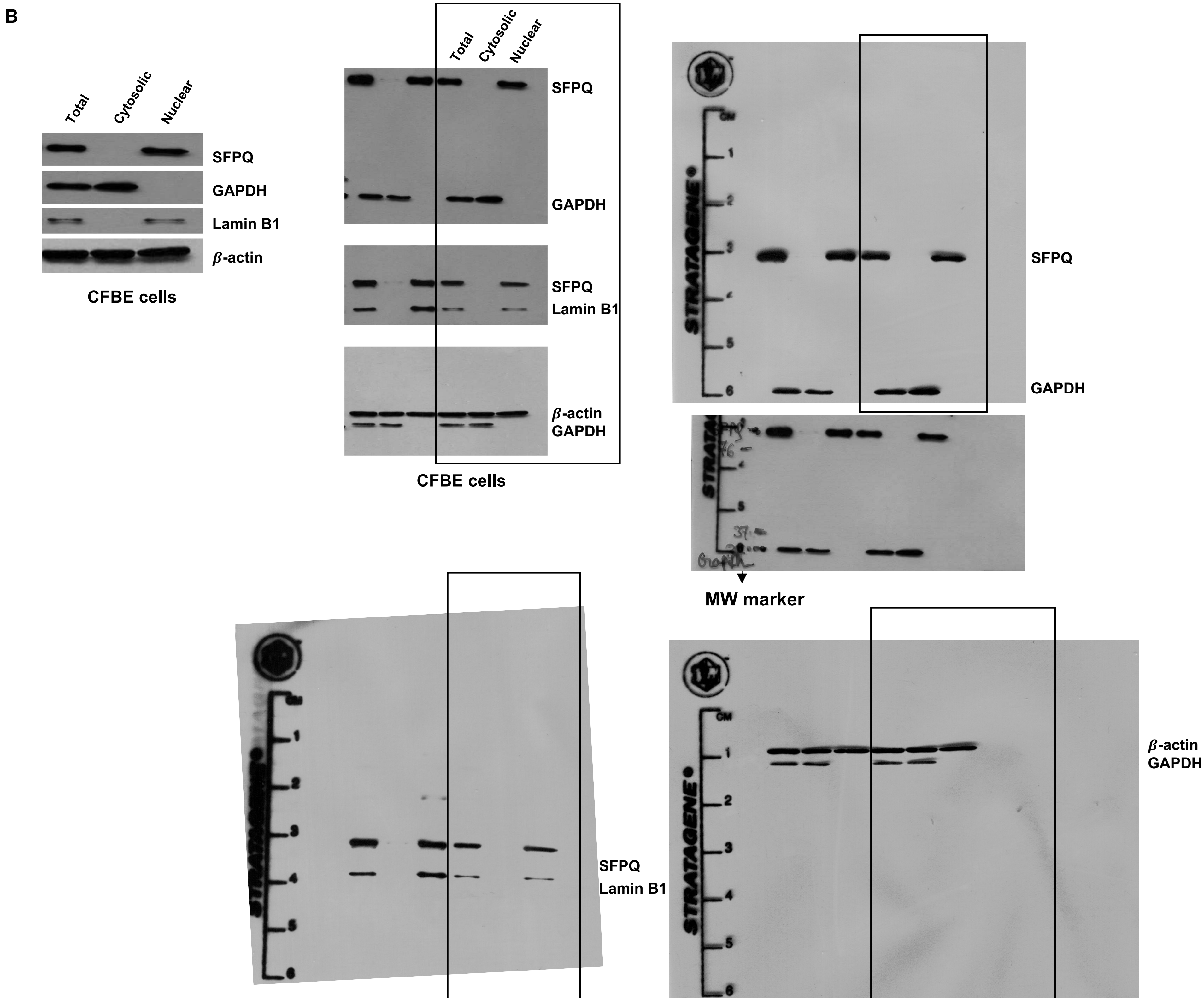

Figure S1: Expression of SFPQ protein in CF lung cells. (B) Localization of SFPQ protein was analyzed by immunoblot CFBE cells containing the F508del mutation. The controls are indicated. The same blots were probed with multiple antibodies for normalization (endogenous controls) as well as subcellular fractionation (GAPDH is in the cytosolic fraction while Lamin B1 is exclusive to the nuclear fraction) and hence include the multiple exposures. The molecular weight markers are indicated.

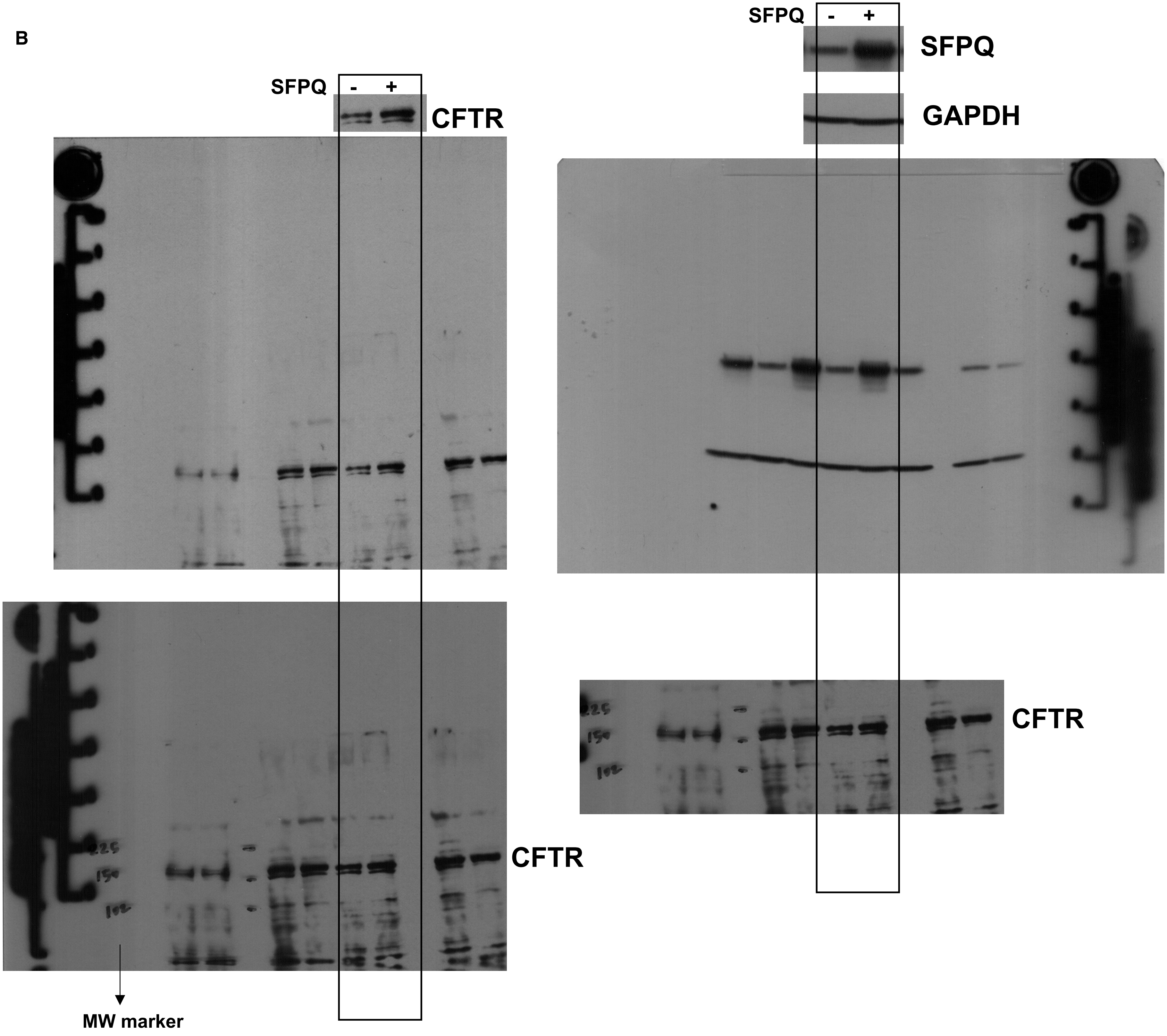

Figure S2: Expression of CFTR protein in CF lung cells. (B) CFTR protein was analyzed by immunoblot in CFBE cells over-expressing SFPQ or control CFBE cells (mock transfected). The same blot was probed with multiple antibodies for normalization (GAPDH antibody) as well as validation of SFPQ expression (SFPQ antibody). Multiple exposures are depicted.

D

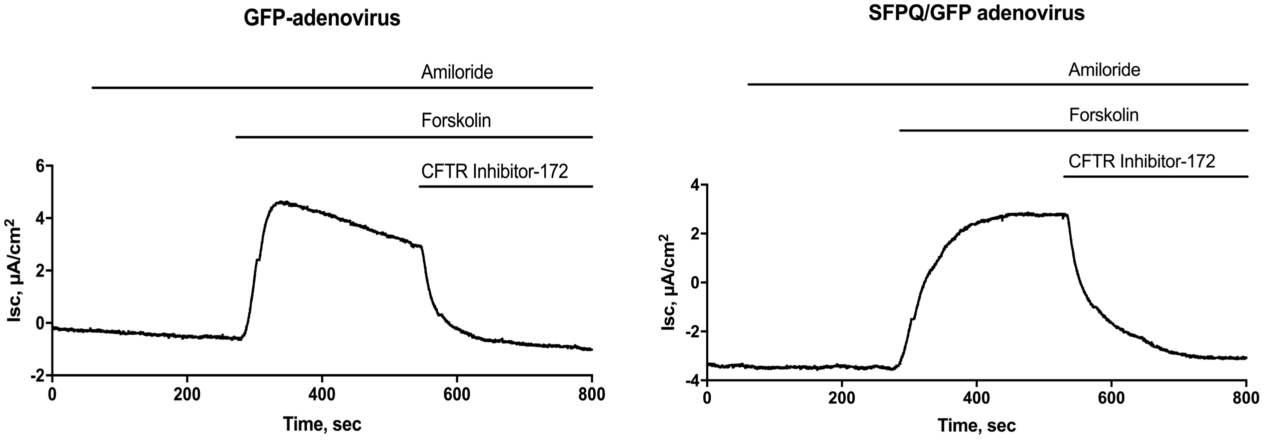

Figure S2: Functional Analyses of F508del-CFTR protein in CF lung cells. (D) The traces for the Ussing Chamber assay are included to indicate functional rescue of F508del-CFTR with over-expression of SFPQ in CF cells.

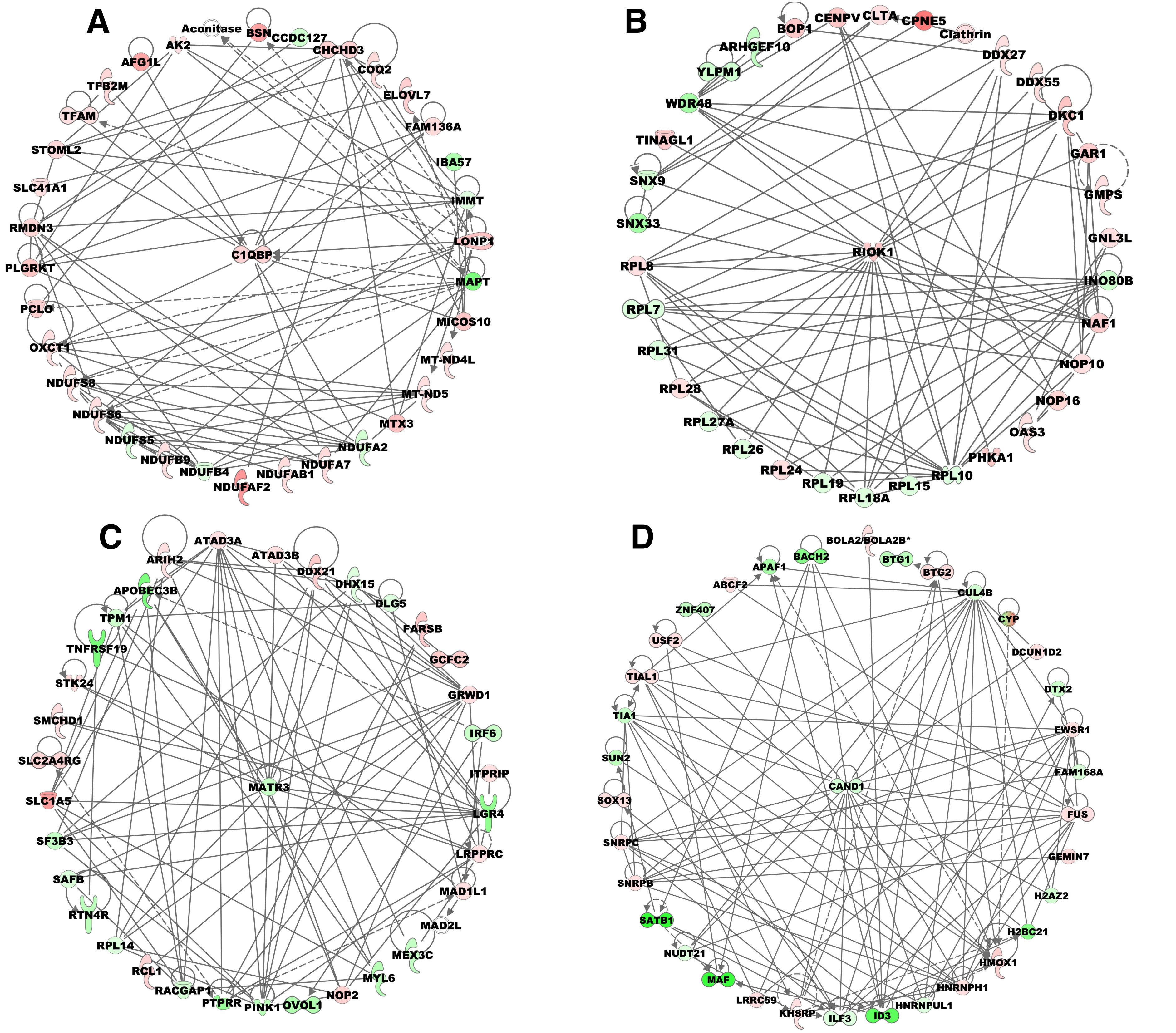

Figure S3. Ingenuity Pathway Analysis gene networks. The radial representations indicate the most enriched networks in SFPQ over-expressed and control CFBE41o- cells. **(A)** Network 5: cell signaling, post-translational modification, protein synthesis; **(B)** Network 6: protein synthesis, RNA damage and repair, RNA post-transcriptional modification; **(C)** Network 7: cell cycle, organismal injury and abnormalities, RNA post-transcriptional modification; **(D)** Network 8: cell cycle, post-translational modification, RNA post-transcriptional modification. Green color denotes down regulation, red color denotes upregulation whereas grey color denotes those present within our data set but not changed significantly. Solid arrows represent the genes that interact directly, while dotted arrows represent indirect interactions between genes.

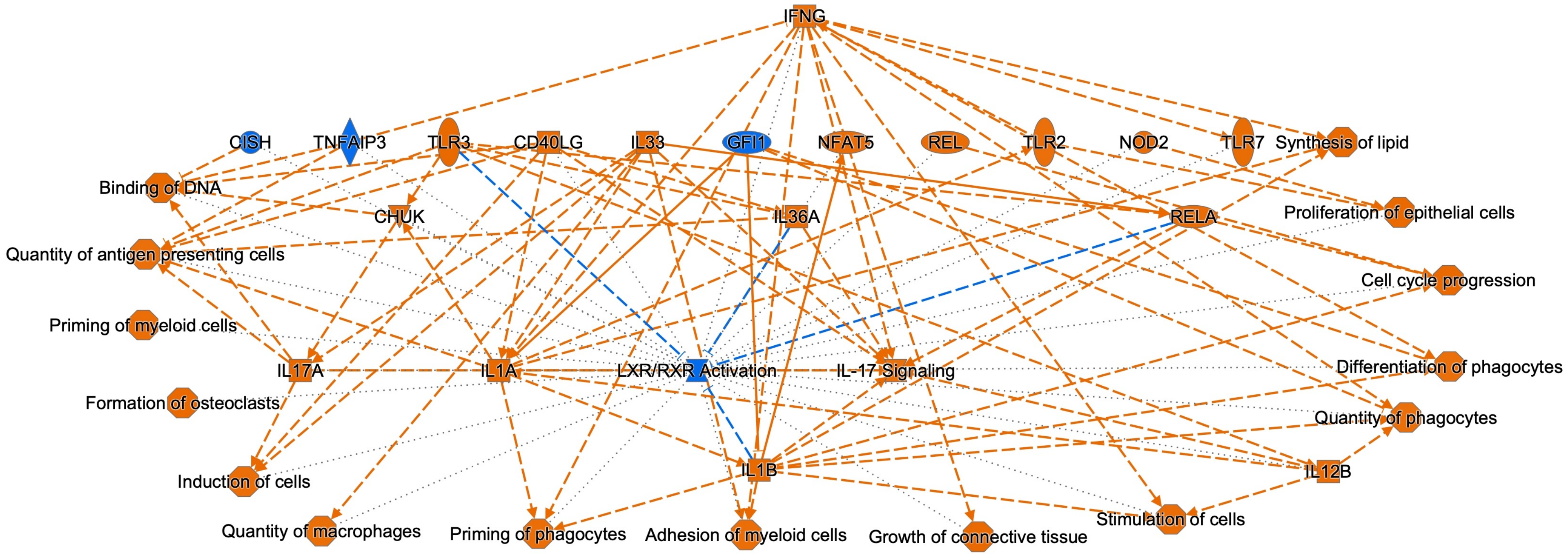

Figure S4. Graphical summary. The predicted key functions that are altered in SFPQ overexpressed versus control CFBE41o^{-} cells are indicated. Orange circles represent predicted activation of functions, blue circles represent predicted inhibition of functions. Solid arrows represent the genes that interact directly, while dotted arrows represent indirect interactions between genes.

Supplementary Table1.

Supplementary Table1A. List of significant up and down genes

Gene_Name	Description	log2FoldChange	P-value	P-adj
MUC20	mucin 20	-1.01039616	9.73E-48	1.24E-45
AC069213.1		-1.029637003	1.10E-24	5.96E-23
TMEM71	transmembrane protein 71 [Source:HGNC Symbol;Acc:26572]	-1.066398818	1.72E-03	8.11E-03
ARID5B	AT rich interactive domain 5B (MRF1-like) [Source:HGNC Symbol;Acc:17362]	-1.066854938	4.33E-56	7.33E-54
MIR4800	microRNA 4800 [Source:HGNC Symbol;Acc:41877]	-1.110265449	4.30E-03	1.78E-02
DACT1	dishevelled-binding antagonist of beta-catenin 1 [Source:HGNC Symbol;Acc:17748]	-1.121894207	1.18E-10	2.01E-09
RP1-140K8.5		-1.130298391	4.69E-07	4.90E-06
LRRC3DN	LRRC3 downstream neighbor (non-protein coding) [Source:HGNC Symbol;Acc:1270]	-1.131460168	1.95E-04	1.21E-03
ACTBL2	actin	-1.137563817	4.81E-05	3.44E-04
DIO2	deiodinase	-1.139971727	2.87E-24	1.49E-22
EGR1	early growth response 1 [Source:HGNC Symbol;Acc:3238]	-1.154900437	1.11E-30	8.02E-29
FILIP1L	filamin A interacting protein 1-like [Source:HGNC Symbol;Acc:24589]	-1.168467692	7.05E-50	9.92E-48
FBXO32	F-box protein 32 [Source:HGNC Symbol;Acc:16731]	-1.182025423	1.14E-52	1.84E-50
RGMA	repulsive guidance molecule family member a [Source:HGNC Symbol;Acc:30308]	-1.254188055	5.98E-05	4.21E-04
KCND2	potassium voltage-gated channel	-1.296953915	2.26E-03	1.03E-02
C10orf10	chromosome 10 open reading frame 10 [Source:HGNC Symbol;Acc:23355]	-1.31859787	8.69E-17	2.80E-15
NKAIN4	Na+/K+ transporting ATPase interacting 4 [Source:HGNC Symbol;Acc:16191]	-1.323484717	2.17E-30	1.54E-28
ST8SIA2	ST8 alpha-N-acetyl-neuraminide alpha-2	-1.34537211	4.82E-04	2.69E-03
GBP2	guanylate binding protein 2	-1.4006592	1.47E-16	4.68E-15
PIK3IP1	phosphoinositide-3-kinase interacting protein 1 [Source:HGNC Symbol;Acc:24942]	-1.412464462	2.42E-15	6.87E-14
LGR5	leucine-rich repeat containing G protein-coupled receptor 5 [Source:HGNC Symbol;Acc:4504]	-1.799523007	8.31E-84	2.87E-81
UBD	ubiquitin D [Source:HGNC Symbol;Acc:18795]	-2.014123415	1.17E-22	5.63E-21
GH1	growth hormone 1 [Source:HGNC Symbol;Acc:4261]	11.25216684	1.52E-21	6.87E-20
SFPQ	splicing factor proline/glutamine-rich [Source:HGNC Symbol;Acc:10774]	4.020463412	0.00E+00	0.00E+00
NPC1L1	NPC1-like 1 [Source:HGNC Symbol;Acc:7898]	3.822161138	2.19E-12	4.53E-11
HSPA6	heat shock 70kDa protein 6 (HSP70B') [Source:HGNC Symbol;Acc:5239]	2.551635306	8.51E-17	2.75E-15

AOC3	amine oxidase	2.545859643	1.71E-09	2.58E-08
CTB-119C2.1		2.095450496	9.94E-08	1.16E-06
BHLHA15	basic helix-loop-helix family	1.783483069	1.55E-05	1.23E-04
IL1A	interleukin 1	1.760130911	2.37E-05	1.82E-04
CMYA5	cardiomyopathy associated 5 [Source:HGNC Symbol;Acc:14305]	1.598531684	1.23E-09	1.89E-08
BCYRN1	brain cytoplasmic RNA 1 [Source:HGNC Symbol;Acc:1022]	1.503946679	3.10E-04	1.82E-03
GDF15	growth differentiation factor 15 [Source:HGNC Symbol;Acc:30142]	1.495407909	1.79E-26	1.04E-24
CHAC1	ChaC	1.486138838	1.72E-64	3.95E-62
ELFN1	extracellular leucine-rich repeat and fibronectin type III domain containing 1 [Source:HGNC Symbol;Acc:33154]	1.467537752	1.11E-12	2.36E-11
ABCA1	ATP-binding cassette	1.390407877	4.88E-37	4.38E-35
PPP1R32	protein phosphatase 1	1.353480543	1.34E-03	6.54E-03
TNF	tumor necrosis factor [Source:HGNC Symbol;Acc:11892]	1.322082546	1.05E-03	5.31E-03
GEM	GTP binding protein overexpressed in skeletal muscle [Source:HGNC Symbol;Acc:4234]	1.308561367	6.10E-04	3.31E-03
ABCG1	ATP-binding cassette	1.24513175	9.82E-06	8.12E-05
PSTPIP1	proline-serine-threonine phosphatase interacting protein 1 [Source:HGNC Symbol;Acc:9580]	1.23668172	1.95E-03	9.01E-03
AC226119.5		1.221695029	1.79E-04	1.13E-03
SLC16A6	solute carrier family 16	1.196287703	2.65E-04	1.58E-03
ADM2	adrenomedullin 2 [Source:HGNC Symbol;Acc:28898]	1.170821341	3.72E-36	3.23E-34
KLHDC7B	kelch domain containing 7B [Source:HGNC Symbol;Acc:25145]	1.168650669	4.60E-08	5.73E-07
SNAI1	snail family zinc finger 1 [Source:HGNC Symbol;Acc:11128]	1.168277821	7.74E-05	5.31E-04
SLAMF7	SLAM family member 7 [Source:HGNC Symbol;Acc:21394]	1.155005196	1.60E-03	7.63E-03
MYH3	myosin	1.141833628	4.50E-07	4.71E-06
ASNSP1	asparagine synthetase pseudogene 1 [Source:HGNC Symbol;Acc:754]	1.108905423	4.93E-08	6.10E-07
RP11-791G15.2		1.097644222	1.44E-04	9.30E-04
FLG	filaggrin [Source:HGNC Symbol;Acc:3748]	1.086281372	1.42E-03	6.88E-03
ADAMTS17	ADAM metallopeptidase with thrombospondin type 1 motif	1.080898814	2.03E-03	9.31E-03
SERPINB10	serpin peptidase inhibitor	1.075627577	2.49E-21	1.12E-19
AURKC	aurora kinase C [Source:HGNC Symbol;Acc:11391]	1.074716149	7.85E-03	2.98E-02
SPRY4	sprouty homolog 4 (Drosophila) [Source:HGNC Symbol;Acc:15533]	1.063418819	2.93E-40	2.95E-38
SPG20OS	SPG20 opposite strand [Source:HGNC Symbol;Acc:39933]	1.058585338	7.12E-03	2.75E-02
C7orf61	chromosome 7 open reading frame 61 [Source:HGNC Symbol;Acc:22135]	1.057977468	6.91E-03	2.68E-02
LTA	lymphotoxin alpha [Source:HGNC Symbol;Acc:6709]	1.052936147	2.41E-04	1.46E-03

SERPINB2	serpin peptidase inhibitor	1.035783883	2.12E-43	2.38E-41
SLC7A11	solute carrier family 7 (anionic amino acid transporter light chain	1.021532237	1.58E-82	5.35E-80
DDIT4	DNA-damage-inducible transcript 4 [Source:HGNC Symbol;Acc:24944]	1.017150037	2.79E-209	5.15E-206
SLC16A14	solute carrier family 16	1.005927465	7.56E-09	1.05E-07
GABRR2	gamma-aminobutyric acid (GABA) A receptor	1.004448791	6.41E-03	2.50E-02
IL8	interleukin 8 [Source:HGNC Symbol;Acc:6025]	1.002567434	2.43E-03	1.09E-02
CLGN	calmegin [Source:HGNC Symbol;Acc:2060]	0.998116821	1.14E-02	4.08E-02
HOXB8	homeobox B8 [Source:HGNC Symbol;Acc:5119]	0.996583106	3.30E-04	1.93E-03

Supplementary Table1B. List of genes associated with TP53 regulatory molecule.

Symbol	Entrez Gene Name	Expr Log Ratio
CDKN1A	cyclin dependent kinase inhibitor 1A	0.350032468
CTNNB1	catenin beta 1	-0.215240719
E2f	--	
E2F4	E2F transcription factor 4	0.115710421
HIF1A	hypoxia inducible factor 1 subunit alpha	0.109647773
IFNG	interferon gamma	
IRF1	interferon regulatory factor 1	0.17586281
JUN	Jun proto-oncogene, AP-1 transcription factor subunit	-0.302380982
MAX	MYC associated factor X	
MLXIPL	MLX interacting protein like	0.371322695
MYC	MYC proto-oncogene, bHLH transcription factor	0.270412782
NFkB (complex)	--	
NFKBIA	NFKB inhibitor alpha	-0.252985537
Rb	--	
RB1	RB transcriptional corepressor 1	
RBL1	RB transcriptional corepressor like 1	0.187188349
RELA	RELA proto-oncogene, NF-kB subunit	
SP1	Sp1 transcription factor	
STAT1	signal transducer and activator of transcription 1	
STAT3	signal transducer and activator of transcription 3	-0.096472127
TNF	tumor necrosis factor	1.322082546
TP53	tumor protein p53	-0.16354502
TP63	tumor protein p63	
TP73	tumor protein p73	-0.183199108

Supplementary Table1C. List of genes associated with 8-bromo-cAMP regulatory molecule.

Symbol	Entrez Gene Name	Expr Log Ratio
8-bromo-cAMP	--	
Ap1	--	
beta-estradiol	--	
cyclic AMP	--	
EGR1	early growth response 1	-1.154900437
ESR1	estrogen receptor 1	
ESR2	estrogen receptor 2	
HDAC1	histone deacetylase 1	-0.131604382
IL6	interleukin 6	0.570681249
JUN	Jun proto-oncogene, AP-1 transcription factor subunit	-0.302380982
NFkB (complex)	--	
NFKBIA	NFKB inhibitor alpha	-0.252985537
RELA	RELA proto-oncogene, NF-kB subunit	
SP1	Sp1 transcription factor	
STAT3	signal transducer and activator of transcription 3	-0.096472127
TNF	tumor necrosis factor	1.322082546
TP53	tumor protein p53	-0.16354502

Supplementary Table1D. List of genes associated with SIRT1regulatory molecule.

Symbol	Entrez Gene Name	Expr Log Ratio
FOXO1	forkhead box O1	
IFNG	interferon gamma	
IGF1	insulin like growth factor 1	
Insulin	--	
JUN	Jun proto-oncogene, AP-1 transcription factor subunit	-0.302380982
MYC	MYC proto-oncogene, bHLH transcription factor	0.270412782
NFKB1	nuclear factor kappa B subunit 1	
NFkB (complex)	--	
NFKBIA	NFKB inhibitor alpha	-0.252985537
PPARA	peroxisome proliferator activated receptor alpha	
PPARG	peroxisome proliferator activated receptor gamma	
PPARGC1A	PPARG coactivator 1 alpha	
RELA	RELA proto-oncogene, NF-kB subunit	
SIRT1	sirtuin 1	-0.187677809
SP1	Sp1 transcription factor	
STAT1	signal transducer and activator of transcription 1	
STAT3	signal transducer and activator of transcription 3	-0.096472127
TNF	tumor necrosis factor	1.322082546
TP53	tumor protein p53	-0.16354502

Supplementary Table1E. List of DEG associated with SFPQ and CFTR

Symbol	Entrez Gene Name	Expr Log Ratio
26s Proteasome	--	
AHSA1	activator of HSP90 ATPase activity 1	0.128738186
AP1B1	adaptor related protein complex 1 subunit beta 1	0.099411871
AP2M1	adaptor related protein complex 2 subunit mu 1	-0.073697623
CEBPB	CCAAT enhancer binding protein beta	0.524058422
CTNNB1	catenin beta 1	-0.215240719
EGFR	epidermal growth factor receptor	0.223517234
HUWE1	HECT, UBA and WWE domain containing E3 ubiquitin protein ligase 1	-0.169190827
KAT5	lysine acetyltransferase 5	-0.147105869
LMNA	lamin A/C	-0.06881414
MOV10	Mov10 RISC complex RNA helicase	0.124592479
PAWR	pro-apoptotic WT1 regulator	-0.165355672
POT1	protection of telomeres 1	-0.138270722
PRKDC	protein kinase, DNA-activated, catalytic subunit	-0.045593954
PRRC2C	proline rich coiled-coil 2C	-0.108700541
PSAP	prosaposin	-0.184526752
PSMA3	proteasome 20S subunit alpha 3	0.085475663
PTGS2	prostaglandin-endoperoxide synthase 2	0.818443077
RTRAF	RNA transcription, translation and transport factor	0.148525056
SFN	stratifin	-0.207535542
SFPQ	splicing factor proline and glutamine rich	4.020463412
SMARCC2	SWI/SNF related, matrix associated, actin dependent regulator of chromatin subfamily c member 2	-0.146229582
SRRM1	serine and arginine repetitive matrix 1	0.12754636
TNF	tumor necrosis factor	1.322082546
TP53	tumor protein p53	-0.16354502
TP53BP1	tumor protein p53 binding protein 1	-0.233685387
UBC	ubiquitin C	-0.069393422
VCL	vinculin	-0.335355963

Supplementary Table 2.

Supplementary Table2A. List of genes involved in network 1.

Symbol	Entrez Gene Name	Expr Log Ratio
AIDA	axin interactor, dorsalization associated	0.130590501
ANXA8/ANXA8L1	annexin A8 like 1	-0.212708923
APOBEC3A	apolipoprotein B mRNA editing enzyme catalytic subunit 3A	-0.686824499
DAZAP1	DAZ associated protein 1	0.165410366
DAZAP2	DAZ associated protein 2	-0.175120887
DDX19B	DEAD-box helicase 19B	0.139957884
DHX32	DEAH-box helicase 32 (putative)	-0.168344869
FAM118A	family with sequence similarity 118 member A	0.14258255
GOLGA8K (includes others)	golgin A8 family member K	0.566859107
HEBP1	heme binding protein 1	0.231578399
KCTD10	potassium channel tetramerization domain containing 10	-0.237521859
KLHDC2	kelch domain containing 2	-0.435191666
KLHDC3	kelch domain containing 3	-0.317577334
LAPTM4A	lysosomal protein transmembrane 4 alpha	-0.264290145
MAPRE2	microtubule associated protein RP/EB family member 2	-0.305466674
MINDY1	MINDY lysine 48 deubiquitinase 1	-0.412957135
MINDY2	MINDY lysine 48 deubiquitinase 2	0.193295029
MOCOS	molybdenum cofactor sulfurase	0.248883041
NEDD8	NEDD8 ubiquitin like modifier	0.26185969
NEDD4L	NEDD4 like E3 ubiquitin protein ligase	-0.125833495
PLSCR4	phospholipid scramblase 4	-0.353485613
PRRG1	proline rich and Gla domain 1	-0.294319696
RBM12	RNA binding motif protein 12	-0.177982379
RNF145	ring finger protein 145	-0.284517158
RNF185	ring finger protein 185	0.138614719
SHKBP1	SH3KBP1 binding protein 1	-0.131534469
SMIM14	small integral membrane protein 14	-0.718632472
SMURF2	SMAD specific E3 ubiquitin protein ligase 2	0.132364114
TENT5A	terminal nucleotidyltransferase 5A	-0.191764994
TMEM127	transmembrane protein 127	-0.169528889
TRIP12	thyroid hormone receptor interactor 12	-0.069103806
TTC3	tetratricopeptide repeat domain 3	-0.175278774
UBB	ubiquitin B	-0.056862464
UBC	ubiquitin C	-0.069393422
UBE4B	ubiquitination factor E4B	-0.186508766

Supplementary Table2B. List of genes involved in network 2.

Symbol	Entrez Gene Name	Expr Log Ratio
ATP5F1B	ATP synthase F1 subunit beta	0.080534505
C1orf35	chromosome 1 open reading frame 35	0.172289491
C6orf47	chromosome 6 open reading frame 47	-0.180217845
CACFD1	calcium channel flower domain containing 1	-0.291068767
DERL3	derlin 3	-0.360446176
DLG1	discs large MAGUK scaffold protein 1	0.084148839
DTNA	dystrobrevin alpha	-0.407785588
EAF1	ELL associated factor 1	0.164090682
EIF5A2	eukaryotic translation initiation factor 5A2	0.420041016
FAM163A	family with sequence similarity 163 member A	-0.424890155
FRZB	frizzled related protein	0.652850812
FYCO1	FYVE and coiled-coil domain autophagy adaptor 1	-0.194362526
FZD7	frizzled class receptor 7	-0.361691304
GPRC5B	G protein-coupled receptor class C group 5 member B	-0.443946362
HMGB3	high mobility group box 3	-0.268387151
IER5	immediate early response 5	0.159959088
KCNJ12	potassium inwardly rectifying channel subfamily J member 12	0.775830765
MAL2	mal, T cell differentiation protein 2 (gene/pseudogene)	0.149393659
MRFAP1L1	Morf4 family associated protein 1 like 1	-0.13835192
NDRG4	NDRG family member 4	-0.736271942
PLD3	phospholipase D family member 3	-0.158205816
PTS	6-pyruvoyltetrahydropterin synthase	0.340264896
RAB30	RAB30, member RAS oncogene family	-0.3063478
RABAC1	Rab acceptor 1	-0.186529804
RETREG3	reticulophagy regulator family member 3	-0.417460495
SDCBP	syndecan binding protein	-0.238512119
SEC63	SEC63 homolog, protein translocation regulator	0.260530287
SESTD1	SEC14 and spectrin domain containing 1	-0.231503826
SLC25A22	solute carrier family 25 member 22	0.29096108
SLC39A8	solute carrier family 39 member 8	0.16327346
TMEM126B	transmembrane protein 126B	0.213388394
VANGL2	VANGL planar cell polarity protein 2	-0.219975587
ZC3H8	zinc finger CCCH-type containing 8	-0.16797602
ZC3H13	zinc finger CCCH-type containing 13	0.428720825
ZMYM3	zinc finger MYM-type containing 3	-0.191665091

Supplementary Table2C. List of genes involved in network 3.

Symbol	Entrez Gene Name	Expr Log Ratio
AURKAIP1	aurora kinase A interacting protein 1	0.168300292
CCDC59	coiled-coil domain containing 59	0.221781251
CENPA	centromere protein A	-0.188184144
CENPC	centromere protein C	-0.280850825
CENPI	centromere protein I	-0.321340949
CSF2	colony stimulating factor 2	0.811309261
DAP3	death associated protein 3	0.165125656
DDX51	DEAD-box helicase 51	0.165977095
Glutathione-S-transferase	--	
HJURP	Holliday junction recognition protein	-0.337522593
MIS18BP1	MIS18 binding protein 1	-0.264819182
MRPL3	mitochondrial ribosomal protein L3	0.231829458
MRPL58	mitochondrial ribosomal protein L58	0.235751603
MRPS9	mitochondrial ribosomal protein S9	0.206459287
MRPS10	mitochondrial ribosomal protein S10	-0.231328906
MRPS14	mitochondrial ribosomal protein S14	0.230413165
MRPS23	mitochondrial ribosomal protein S23	0.174510785
MRPS25	mitochondrial ribosomal protein S25	0.287662832
MRPS26	mitochondrial ribosomal protein S26	0.102258463
MRPS27	mitochondrial ribosomal protein S27	-0.177342255
MRPS34	mitochondrial ribosomal protein S34	0.121211081
MRPS35	mitochondrial ribosomal protein S35	0.139956786
MRPS18C	mitochondrial ribosomal protein S18C	0.160859742
NOL8	nucleolar protein 8	0.128891221
NOL12	nucleolar protein 12	0.108627855
NUAK2	NUAK family kinase 2	-0.18676308
PDF	peptide deformylase, mitochondrial	0.274681429
RBM34	RNA binding motif protein 34	0.222778211
RGL2	ral guanine nucleotide dissociation stimulator like 2	-0.378736952
RSBN1	round spermatid basic protein 1	-0.282516269
TRA2A	transformer 2 alpha homolog	0.243184676
URB2	URB2 ribosome biogenesis homolog	0.133519287
UTP11	UTP11 small subunit processome component	0.206236566
ZNF3	zinc finger protein 3	-0.283920798
ZNF770	zinc finger protein 770	0.132075816

Supplementary Table2D. List of genes involved in network 4.

Symbol	Entrez Gene Name	Expr Log Ratio
AOX1	aldehyde oxidase 1	-0.70959354
ARHGAP21	Rho GTPase activating protein 21	-0.15997484
ARID5A	AT-rich interaction domain 5A	-0.36465654
ARL8A	ADP ribosylation factor like GTPase 8A	0.27358335
BATF	basic leucine zipper ATF-like transcription factor	-0.21990083
CITED2	Cbp/p300 interacting transactivator with Glu/Asp rich carboxy-terminal domain 2	-0.443646
CMPK2	cytidine/uridine monophosphate kinase 2	0.357061293
CPSF1	cleavage and polyadenylation specific factor 1	-0.09690852
CPSF4	cleavage and polyadenylation specific factor 4	0.122825575
CSTF2	cleavage stimulation factor subunit 2	0.209139565
FAM168B	family with sequence similarity 168 member B	0.126295105
GLRB	glycine receptor beta	0.422890307
Glycine Receptor	--	
HHEX	hematopoietically expressed homeobox	0.441111546
KMT2C	lysine methyltransferase 2C	-0.22865039
LAMP3	lysosomal associated membrane protein 3	0.365956108
MOSPD3	motile sperm domain containing 3	-0.48204572
NEK6	NIMA related kinase 6	-0.25781115
NEK7	NIMA related kinase 7	-0.15007808
NEK9	NIMA related kinase 9	-0.12413397
NOP53	NOP53 ribosome biogenesis factor	-0.15195533
PHF14	PHD finger protein 14	-0.15472582
PLK4	polo like kinase 4	-0.1655475
PPP1R32	protein phosphatase 1 regulatory subunit 32	1.353480543
RBAK	RB associated KRAB zinc finger	0.159478153
RBL1	RB transcriptional corepressor like 1	0.187188349
RILPL1	Rab interacting lysosomal protein like 1	0.437475897
SASH1	SAM and SH3 domain containing 1	0.189038904
SYMPK	symplekin	-0.15270304
TLR4	toll like receptor 4	0.393732288
TSEN15	tRNA splicing endonuclease subunit 15	0.371270808
TSEN34	tRNA splicing endonuclease subunit 34	-0.21145809
WDR33	WD repeat domain 33	-0.12267053
ZC3H14	zinc finger CCCH-type containing 14	0.158326068
ZNF121	zinc finger protein 121	0.23879196

Supplementary Table2E. List of genes involved in network 5.

Symbol	Entrez Gene Name	Expr Log Ratio
Aconitase	--	
AFG1L	AFG1 like ATPase	0.493315038
AK2	adenylate kinase 2	0.117335483
BSN	bassoon presynaptic cytomatrix protein	0.510297292
C1QBP	complement C1q binding protein	0.232362844
CCDC127	coiled-coil domain containing 127	-0.208913175
CHCHD3	coiled-coil-helix-coiled-coil-helix domain containing 3	0.244755715
COQ2	coenzyme Q2, polyprenyltransferase	0.197203759
ELOVL7	ELOVL fatty acid elongase 7	0.277241327
FAM136A	family with sequence similarity 136 member A	0.196776782
IBA57	iron-sulfur cluster assembly factor IBA57	-0.330651671
IMMT	inner membrane mitochondrial protein	-0.082462673
LONP1	lon peptidase 1, mitochondrial	0.334958386
MAPT	microtubule associated protein tau	-0.560002795
MICOS10	mitochondrial contact site and cristae organizing system subunit 10	0.307686548
MT-ND5	NADH dehydrogenase, subunit 5 (complex I)	0.138487827
MT-ND4L	NADH dehydrogenase, subunit 4L (complex I)	0.111595448
MTX3	metaxin 3	0.338592213
NDUFA2	NADH:ubiquinone oxidoreductase subunit A2	-0.187729691
NDUFA7	NADH:ubiquinone oxidoreductase subunit A7	0.201587411
NDUFAB1	NADH:ubiquinone oxidoreductase subunit AB1	0.189968851
NDUFAF2	NADH:ubiquinone oxidoreductase complex assembly factor 2	0.574563943
NDUFB4	NADH:ubiquinone oxidoreductase subunit B4	-0.115315284
NDUFB9	NADH:ubiquinone oxidoreductase subunit B9	0.107704106
NDUFS5	NADH:ubiquinone oxidoreductase subunit S5	-0.146751615
NDUFS6	NADH:ubiquinone oxidoreductase subunit S6	0.134170392
NDUFS8	NADH:ubiquinone oxidoreductase core subunit S8	0.18215951
OXCT1	3-oxoacid CoA-transferase 1	0.127538129
PCLO	piccolo presynaptic cytomatrix protein	0.256933947
PLGRKT	plasminogen receptor with a C-terminal lysine	0.321625139
RMDN3	regulator of microtubule dynamics 3	0.227576868
SLC41A1	solute carrier family 41 member 1	0.141268684
STOML2	stomatin like 2	0.211058908
TFAM	transcription factor A, mitochondrial	0.073852245
TFB2M	transcription factor B2, mitochondrial	0.214787876

Supplementary Table2F. List of genes involved in network 6.

Symbol	Entrez Gene Name	Expr Log Ratio
ARHGEF10	Rho guanine nucleotide exchange factor 10	-0.260735559
BOP1	BOP1 ribosomal biogenesis factor	0.302172602
CENPV	centromere protein V	0.32013917
Clathrin	--	
CLTA	clathrin light chain A	0.075910478
CPNE5	copine 5	0.75731273
DDX27	DEAD-box helicase 27	0.110716753
DDX55	DEAD-box helicase 55	0.183774115
DKC1	dyskerin pseudouridine synthase 1	0.320837659
GAR1	GAR1 ribonucleoprotein	0.274570793
GMPS	guanine monophosphate synthase	0.159087002
GNL3L	G protein nucleolar 3 like	0.131827019
INO80B	INO80 complex subunit B	-0.215493787
NAF1	nuclear assembly factor 1 ribonucleoprotein	0.272625907
NOP10	NOP10 ribonucleoprotein	0.164327992
NOP16	NOP16 nucleolar protein	0.230874108
OAS3	2'-5'-oligoadenylate synthetase 3	0.152105699
PHKA1	phosphorylase kinase regulatory subunit alpha 1	0.288911662
RIOK1	RIO kinase 1	0.303942924
RPL7	ribosomal protein L7	-0.080051973
RPL8	ribosomal protein L8	-0.099844874
RPL10	ribosomal protein L10	-0.082889826
RPL15	ribosomal protein L15	-0.052786916
RPL19	ribosomal protein L19	0.071393988
RPL24	ribosomal protein L24	-0.055720217
RPL26	ribosomal protein L26	-0.070764264
RPL28	ribosomal protein L28	0.087786566
RPL31	ribosomal protein L31	-0.068088799
RPL18A	ribosomal protein L18a	-0.104306129
RPL27A	ribosomal protein L27a	0.059212796
SNX9	sorting nexin 9	-0.379712806
SNX33	sorting nexin 33	-0.185730585
TINAGL1	tubulointerstitial nephritis antigen like 1	0.277921586
WDR48	WD repeat domain 48	-0.375354284
YLPM1	YLP motif containing 1	-0.198191638

Supplementary Table2G. List of genes involved in network 7.

Symbol	Entrez Gene Name	Expr Log Ratio
APOBEC3B	apolipoprotein B mRNA editing enzyme catalytic subunit 3B	-0.557040291
ARIH2	ariadne RBR E3 ubiquitin protein ligase 2	0.165757483
ATAD3A	ATPase family AAA domain containing 3A	0.171344947
ATAD3B	ATPase family AAA domain containing 3B	0.154158051
DDX21	DExD-box helicase 21	0.283388748
DHX15	DEAH-box helicase 15	-0.099690277
DLG5	discs large MAGUK scaffold protein 5	-0.118633402
FARSB	phenylalanyl-tRNA synthetase subunit beta	0.303422071
GCFC2	GC-rich sequence DNA-binding factor 2	0.283924589
GRWD1	glutamate rich WD repeat containing 1	0.105633552
IRF6	interferon regulatory factor 6	-0.223609755
ITPRIP	inositol 1,4,5-trisphosphate receptor interacting protein	0.135219704
LGR4	leucine rich repeat containing G protein-coupled receptor 4	-0.47308491
LRPPRC	leucine rich pentatricopeptide repeat containing	0.091249196
MAD1L1	mitotic arrest deficient 1 like 1	0.150056875
MAD2L	--	
MATR3	matrin 3	-0.24189606
MEX3C	mex-3 RNA binding family member C	-0.249695406
MYL6	myosin light chain 6	-0.305498144
NOP2	NOP2 nucleolar protein	0.252293181
OVOL1	ovo like transcriptional repressor 1	-0.328828628
PINK1	PTEN induced kinase 1	-0.30402087
PTPRR	protein tyrosine phosphatase receptor type R	-0.451183299
RACGAP1	Rac GTPase activating protein 1	-0.186424368
RCL1	RNA terminal phosphate cyclase like 1	0.244872758
RPL14	ribosomal protein L14	-0.085979243
RTN4R	reticulon 4 receptor	-0.281898315
SAFB	scaffold attachment factor B	-0.157129246
SF3B3	splicing factor 3b subunit 3	-0.245558895
SLC1A5	solute carrier family 1 member 5	0.555509167
SLC2A4RG	SLC2A4 regulator	0.226906629
SMCHD1	structural maintenance of chromosomes flexible hinge domain containing 1	0.128810987
STK24	serine/threonine kinase 24	0.1944705
TNFRSF19	TNF receptor superfamily member 19	-0.567465395
TPM1	tropomyosin 1	-0.202022602

Supplementary Table2H. List of genes involved in network 8.

Symbol	Entrez Gene Name	Expr Log Ratio
ABCF2	ATP binding cassette subfamily F member 2	0.104928089
APAF1	apoptotic peptidase activating factor 1	-0.375368927
BACH2	BTB domain and CNC homolog 2	-0.508069133
BOLA2/BOLA2B	bolA family member 2	0.172264286
BTG1	BTG anti-proliferation factor 1	-0.284313318
BTG2	BTG anti-proliferation factor 2	0.19596479
CAND1	cullin associated and neddylation dissociated 1	-0.178352199
CUL4B	cullin 4B	-0.188914853
CYP	--	
DCUN1D2	defective in cullin neddylation 1 domain containing 2	0.176281987
DTX2	deltex E3 ubiquitin ligase 2	-0.224263077
EWSR1	EWS RNA binding protein 1	0.100442095
FAM168A	family with sequence similarity 168 member A	-0.161266453
FUS	FUS RNA binding protein	0.168899183
GEMIN7	gem nuclear organelle associated protein 7	0.19954442
H2AZ2	H2A.Z variant histone 2	-0.185113288
H2BC21	H2B clustered histone 21	-0.501661202
HMOX1	heme oxygenase 1	0.261876841
HNRNPH1	heterogeneous nuclear ribonucleoprotein H1	0.067790622
HNRNPUL1	heterogeneous nuclear ribonucleoprotein U like 1	-0.05611029
ID3	inhibitor of DNA binding 3, HLH protein	-0.69362038
ILF3	interleukin enhancer binding factor 3	-0.053153004
KHSRP	KH-type splicing regulatory protein	0.174147752
LRRC59	leucine rich repeat containing 59	0.179381157
MAF	MAF bZIP transcription factor	-0.838184406
NUDT21	nudix hydrolase 21	-0.075760808
SATB1	SATB homeobox 1	-0.865163617
SNRPB	small nuclear ribonucleoprotein polypeptides B and B1	0.111511396
SNRPC	small nuclear ribonucleoprotein polypeptide C	0.122600743
SOX13	SRY-box transcription factor 13	0.143621223
SUN2	Sad1 and UNC84 domain containing 2	-0.288155883
TIA1	TIA1 cytotoxic granule associated RNA binding protein	-0.232282602
TIAL1	TIA1 cytotoxic granule associated RNA binding protein like 1	0.147576362
USF2	upstream transcription factor 2, c-fos interacting	0.157569749
ZNF407	zinc finger protein 407	-0.254008259
